# Modifiable pathways in Alzheimer’s disease: Mendelian randomisation analysis

**DOI:** 10.1136/bmj.j5375

**Published:** 2017-12-07

**Authors:** Susanna C Larsson, Matthew Traylor, Rainer Malik, Martin Dichgans, Stephen Burgess, Hugh S Markus

**Affiliations:** 1 Unit of Nutritional Epidemiology, Institute of Environmental Medicine, Karolinska Institutet, 171 77 Stockholm, Sweden; 2 Stroke Research Group, Department of Clinical Neurosciences, University of Cambridge, Cambridge, UK; 3 Institute for Stroke and Dementia Research, Klinikum der Universität München, Ludwig-Maximilians University, Munich, Germany; 4 Munich Cluster for Systems Neurology (SyNergy), Munich, Germany; 5 German Centre for Neurodegenerative Diseases (DZNE, Munich), Munich, Germany; 6 MRC Biostatistics Unit, University of Cambridge, Cambridge, UK; 7 Department of Public Health and Primary Care, University of Cambridge, Cambridge, UK

## Abstract

**Objective:**

To determine which potentially modifiable risk factors, including socioeconomic, lifestyle/dietary, cardiometabolic, and inflammatory factors, are associated with Alzheimer’s disease.

**Design:**

Mendelian randomisation study using genetic variants associated with the modifiable risk factors as instrumental variables.

**Setting:**

International Genomics of Alzheimer’s Project.

**Participants:**

17 008 cases of Alzheimer’s disease and 37 154 controls.

**Main outcome measures:**

Odds ratio of Alzheimer’s per genetically predicted increase in each modifiable risk factor estimated with Mendelian randomisation analysis.

**Results:**

This study included analyses of 24 potentially modifiable risk factors. A Bonferroni corrected threshold of P=0.002 was considered to be significant, and P<0.05 was considered suggestive of evidence for a potential association. Genetically predicted educational attainment was significantly associated with Alzheimer’s. The odds ratios were 0.89 (95% confidence interval 0.84 to 0.93; P=2.4×10^−6^) per year of education completed and 0.74 (0.63 to 0.86; P=8.0×10^−5^) per unit increase in log odds of having completed college/university. The correlated trait intelligence had a suggestive association with Alzheimer’s (per genetically predicted 1 SD higher intelligence: 0.73, 0.57 to 0.93; P=0.01). There was suggestive evidence for potential associations between genetically predicted higher quantity of smoking (per 10 cigarettes a day: 0.69, 0.49 to 0.99; P=0.04) and 25-hydroxyvitamin D concentrations (per 20% higher levels: 0.92, 0.85 to 0.98; P=0.01) and lower odds of Alzheimer’s and between higher coffee consumption (per one cup a day: 1.26, 1.05 to 1.51; P=0.01) and higher odds of Alzheimer’s. Genetically predicted alcohol consumption, serum folate, serum vitamin B_12_, homocysteine, cardiometabolic factors, and C reactive protein were not associated with Alzheimer’s disease.

**Conclusion:**

These results provide support that higher educational attainment is associated with a reduced risk of Alzheimer’s disease.

## Introduction

Alzheimer’s disease is the leading cause of dementia. The chief hallmarks are amyloid plaques and neurofibrillary tangles.^1^ The amyloid cascade hypothesis implies that accumulation of amyloid β triggers neuronal dysfunction and cell death in the brain.^1^ An alternative theory—the vascular hypothesis—implicates cerebral hypoperfusion as the primary trigger; this drives oxidative stress, deposition of amyloid β, neuroinflammation, blood-brain barrier breakdown, cognitive decline, and neurodegeneration.^2^ ^3^

Apart from increasing age and the apolipoprotein E (APOE) e4 allele, the causes of Alzheimer’s disease are largely unknown, and treatment trials have been disappointing.^4^ This has led to increasing interest in the potential for reducing Alzheimer’s by targeting modifiable risk factors. Conventional observational studies have consistently shown that low educational attainment is associated with an increased risk,^5^ and it has been estimated that 19% of cases are potentially attributable to low education.^6^ Inconclusive evidence from conventional observational studies indicates that obesity, hypertension, and hypercholesterolaemia in midlife and diabetes, smoking, low vitamin D and folate concentrations, hyperhomocysteinaemia, and high C reactive protein concentrations are associated with increased risk, whereas physical activity, a healthy diet, moderate alcohol drinking, and coffee consumption are associated with decreased risk (table A in appendix 1).^5^ ^6^ ^7^ ^8^ ^9^ ^10^ ^11^ A 2010 State of the Science report concluded that there was insufficient evidence to support the association with any modifiable factors with risk.^7^ Available evidence is in large part inadequate as observational studies generally rely on self reported information and are susceptible to confounding and reverse causation bias, and data from randomised trials^12^ ^13^ ^14^ ^15^ ^16^ are scarce and inconclusive.

Mendelian randomisation is a genetic epidemiological method for assessing causal inference by exploiting genetic variants influencing the modifiable risk factor to estimate the unbiased association between the risk factor and risk of disease. Genetic alleles are randomly assorted during conception and thus are less likely to be affected by confounding factors that could bias the observational findings. Furthermore, reverse causation bias is avoided because genotype is not affected by disease. This method is being increasingly used to investigate the potential of different treatment approaches by determining which risk factors are causally associated with disease and therefore might be worth targeting therapeutically.^17^ To decipher potentially causal and modifiable risk factors we applied a Mendelian randomisation approach to examine the associations between multiple potentially modifiable risk factors and Alzheimer’s disease.

## Methods

### Modifiable risk factors

We considered potentially modifiable risk factors that can be grouped under the following categories: socioeconomic, lifestyle/dietary, cardiometabolic, and inflammatory. Within these categories we focused on factors that were identified as having the most consistent evidence for an association with Alzheimer’s disease in meta-analyses of prospective observational studies (table A in appendix 1).^5^ ^8^ ^9^ ^10^ ^11^ We also included intelligence on the basis of its strong genetic correlation with educational attainment^18^ and to increase the number of proxies of cognitive reserve.^19^ ^20^

### Data sources

We performed this analysis with summarised data (effect size estimates and their standard errors) from published genome-wide association studies (fig A in appendix 2). We searched PubMed for genome-wide association studies of the modifiable risk factors and identified genetic variants with genome-wide significant (P <5×10^−8^) associations for educational attainment (years of education completed and college/university completion),^21^ intelligence,^18^ smoking (quantity, initiation, and cessation),^22^ ^23^ alcohol^24^ and coffee^25^ consumption, 25-hydroxyvitamin D (25(OH)D; the primary biomarker of vitamin D status),^26^ serum folate and vitamin B_12_ concentrations,^27^ total homocysteine,^28^ overall obesity (body mass index (BMI)),^29^ abdominal obesity (waist to hip ratio adjusted for BMI),^30^ type 2 diabetes,^31^ fasting glucose and insulin,^32^ systolic and diastolic blood pressure,^33^ blood lipids (high density lipoprotein cholesterol, low density lipoprotein cholesterol, total cholesterol, and triglycerides),^34^ and C reactive protein (a general marker of systemic inflammation).^35^ We did not identify genetic variants with genome-wide significant association with occupation or income (measures of socioeconomic status), physical activity, healthy eating patterns, or vitamins C or E. Details on the risk factor studies from which we obtained summarised data for the current analyses from are presented in table B in appendix 1.

Summarised data for the associations between the genetic variants (that is, the single nucleotide polymorphisms) related to risk factors and Alzheimer’s disease were obtained from the International Genomics of Alzheimer’s Project (IGAP), which has been described elsewhere.^36^ Briefly, this project includes data from 17 008 cases of Alzheimer’s disease and 37 154 controls of European ancestry from four genome-wide association study datasets, including the Alzheimer’s Disease Genetics Consortium (ADGC), the Cohorts for Heart and Aging Research in Genomic Epidemiology consortium (CHARGE), the European Alzheimer’s disease Initiative (EADI), and the Genetic and Environmental Risk in Alzheimer’s disease consortium (GERAD). Details on the original genetic analyses and diagnostic criteria for Alzheimer’s disease are provided in appendix 3. Summarised data for the associations of the genetic variants with the risk factors and Alzheimer’s disease are presented in table C in appendix 1.

### Patient involvement

No patients were involved in the design of the study, recruitment, or conduct of the study. No patients were asked to advise on interpretation or writing up of results. There are no plans to involve patients in dissemination of the results, but results will, after scientific publication, be disseminated to the public in general.

### Genetic variants

For each modifiable risk factor, we selected genetic variants (single nucleotide polymorphisms) associated with the risk factor at thresholds for genome-wide significance (P <5×10^−8^) in the sex combined meta-analysis of the discovery and replication samples of the published genome-wide association studies (table B in appendix 1). We selected only independent genetic variants—that is, not in linkage disequilibrium (defined as r^2^<0.2) with other genetic variants for the same risk factor. When we encountered genetic variants in linkage disequilibrium, we chose the variant with the lowest P value for association with the risk factor. For genetic variants that were not present in IGAP, we used proxies (r^2^>0.9) where available (table B in appendix 1).

### Mendelian randomisation analysis

**Fig 1:**
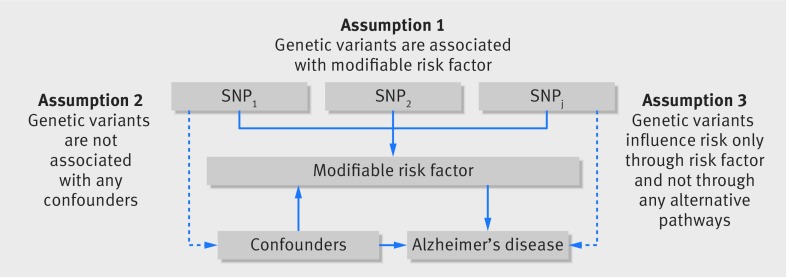
Principles of Mendelian randomisation analysis for modifiable risk factor and risk of Alzheimer’s disease and assumptions that need to be met to obtain unbiased estimates of causal effects. Broken lines represent potential pleiotropic or direct causal effects between variables that would violate Mendelian randomisation assumptions. SNP_1_, SNP_2_, SNP_j_=single nucleotide polymorphisms

The Mendelian randomisation approach we used was based on the following assumptions: the genetic variants used as instrumental variables for the modifiable risk factor are associated with the risk factor; the genetic variants are not associated with any confounders; and the genetic variants are associated with Alzheimer’s through the risk factor only and not through any alternative causal pathway (fig 1).^37^ It also assumed that associations between risk factors and Alzheimer’s are linear with no statistical interactions.

We included analyses of 24 potentially modifiable risk factors. To take into account multiple testing, we used a conservative approach and applied a Bonferroni corrected significance level computed as 0.05 divided by 24 (that is, 0.002). P<0.05 but above the Bonferroni corrected significance threshold was considered as suggestive of evidence for a potential association.

For each genetic variant, we calculated an instrumental variable ratio estimate by dividing the effect size estimate (β coefficient) for the association of the variant with risk of Alzheimer’s by the corresponding estimate for the association of the variant with the modifiable risk factor. In the main analyses, we summarised the ratio estimates for the individual genetic variants using the conventional fixed effect inverse variance weighted method.^38^ For risk factors with a significant or suggestive association with Alzheimer’s, we additionally conducted sensitivity analyses using the weighted median, penalised weighted median, and MR-Egger regression methods.^38^ Pleiotropy was evaluated based on the intercept obtained from the MR-Egger analysis.^37^ ^38^ To investigate the influence of outlying and/or pleiotropic genetic variants, we performed a leave one out analysis in which we omitted one genetic variant in turn.^37^ The strength of the genetic instruments was tested with the F statistic.^39^

Results are presented as odds ratios (95% confidence intervals) per genetically predicted increase in each risk factor. The estimates are scaled by year of education completed, 10 cigarettes a day of smoking, additional drink a week of alcohol consumption, cup a day of coffee consumption, 20% change of 25(OH)D concentrations, and approximate standard deviation (SD) for the other continuous risk factors. For the binary risk factors, the estimates represent the odds ratio per 1 unit higher log odds of the risk factor. All analyses were performed with Stata version 14.2 (StataCorp, College Station, TX) and R version 3.3.3 (R foundation).

## Results

### Education and intelligence

**Fig 2:**
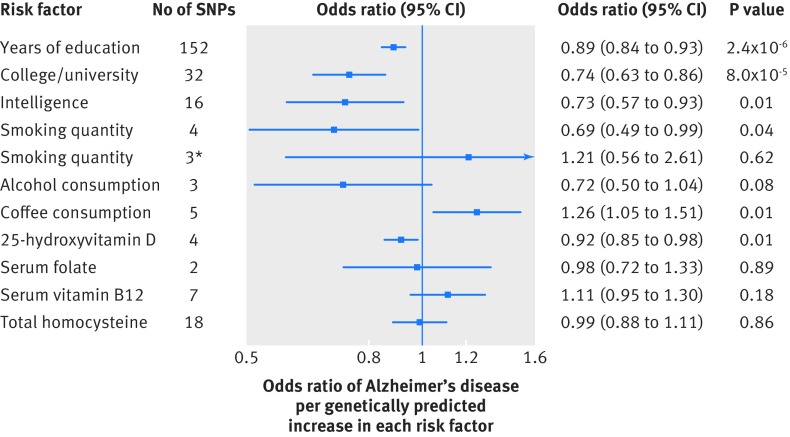
Odds ratios for associations between genetically predicted higher educational attainment, intelligence, and lifestyle and dietary factors and Alzheimer’s disease. Estimates are per year of education completed, 1 unit higher log odds of college/university completion, 1 SD higher intelligence, 10 cigarettes/day, drink of alcohol/week, cup of coffee/day, 20% increase of 25-hydroxyvitamin D concentration, and 1 SD serum folate, serum vitamin B_12_, and total homocysteine. *Excludes one outlying genetic variant (rs1051730) in or near neuronal nicotinic acetylcholine receptor genes (CHRNA3, CHRNA5, and CHRNB4). SNPs=single nucleotide polymorphisms

Genetically predicted higher educational attainment was associated with significantly lower odds of Alzheimer’s disease. The odds ratios were 0.89 (95% confidence interval 0.84 to 0.93; P=2.4×10^−6^) per year of education completed (fig 2) and 0.74 (0.63 to 0.86; P=8.0×10^−5^) per unit higher log odds of having completed college/university (fig 2 and fig B in appendix 2). We found a suggestive association between intelligence and Alzheimer’s. The odds ratio per genetically predicted 1 SD higher intelligence was 0.73 (0.57 to 0.93; P=0.01) (fig 2 and fig C in appendix 2). In leave one out analyses, we found that no single genetic variant had an influential influence on the results for education or intelligence. In addition, the associations were consistent in sensitivity analyses that used the weighted median and penalised weighted median methods but with less precision (fig D in appendix 2). In the MR-Egger analysis, while there was no evidence of directional pleiotropy (all P≥0.11), the precisions of the causal estimates and intercepts were low (fig D in appendix 2). This was mainly because the genetic variants had similar associations with the risk factors and the instrument strength was low for education (F=5.7) but adequate for intelligence (F≥60).

In conventional MR analyses, genetic predisposition towards longer education was associated with lower odds of smoking, fewer cigarettes smoked a day, higher high density lipoprotein cholesterol, lower triglycerides, lower fasting insulin, and lower BMI (P<0.01 for each of these outcomes) (table D in appendix 1). We found no association with systolic or diastolic blood pressure, low density lipoprotein cholesterol, or glucose (all P≥0.35) (table D in appendix 1).

### Lifestyle and dietary factors

There was a suggestive association between genetically predicted higher quantity of smoking and lower odds of Alzheimer’s disease (per 10 cigarettes/day: odds ratio 0.69, 95% confidence interval 0.49 to 0.99; P=0.04) (fig 2 and fig E in appendix 2). The association was driven by a genetic variant (rs1051730) near the nicotinic acetylcholine receptor genes and did not remain when we excluded this variant (1.21, 0.56 to 2.61) (fig 2). Neither initiation (0.71, 0.37 to 1.33; P=0.28) nor cessation (1.16, 0.75 to 1.78; P=0.52) of smoking was associated with Alzheimer’s, but the results were based on a single genetic variant leading to low precision. Genetically predicted alcohol consumption was not associated with Alzheimer’s (fig 2).

We found a suggestive association between genetically predicted higher consumption of coffee and higher odds of Alzheimer’s disease (per cup/day: odds ratio 1.26, 95% confidence interval 1.05 to 1.51; P=0.01) (fig 2 and fig F in appendix 2). In leave one out analysis, the odds ratio ranged from 1.22 (0.95 to 1.56; P=0.11) when we excluded the genetic variant near the CYP1A1 and CYP1A2 gene regions to 1.38 (1.13 to 1.68; P=0.001) when we excluded the variant in POR.

There was a suggestive association between genetically predicted higher 25(OH)D concentrations and lower odds of Alzheimer’s disease (per 20% higher levels: odds ratio 0.92, 95% confidence interval 0.85 to 0.98; P=0.01) (fig 2), and no outlying genetic variant was identified (fig G in appendix 2). Genetically predicted serum folate, serum vitamin B_12_, and total homocysteine concentrations were not associated with AD (fig 2); no single genetic variant had an influential effect on the results.

Results for smoking, coffee consumption, and 25(OH)D were similar in sensitivity analyses that used the weighted median and penalised weighted median methods (fig H in appendix 2). The MR-Egger method showed directional pleiotropy in the smoking analysis (P=0.002) but not in the analyses of coffee (P=0.72) and 25(OH)D (P=0.17). Causal estimates from the method were imprecise in all cases, but there was a suggestive inverse association between smoking and AD (P=0.01) (fig H in appendix 2).

### Cardiometabolic and inflammatory factors

**Fig 3:**
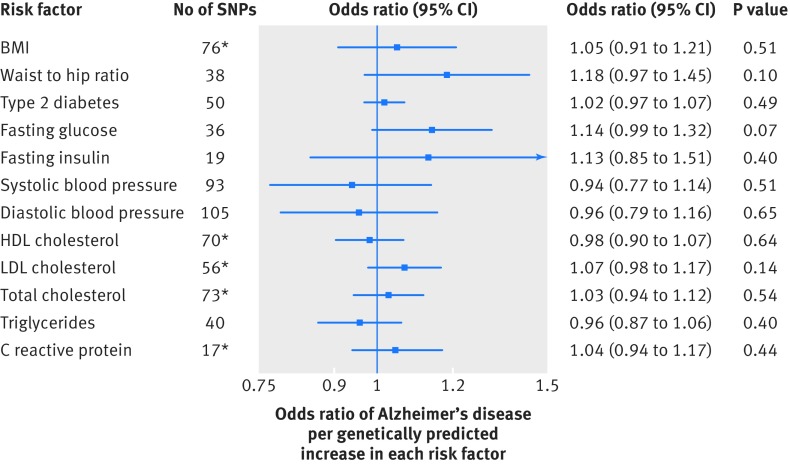
Odds ratios for associations between genetically predicted cardiometabolic and inflammatory factors and Alzheimer’s disease. Estimates are per approximate 1 SD increase of continuous risk factors and per 1 unit higher log odds of type 2 diabetes. *Excludes one pleiotropic genetic variant near the APOE gene (also near APOC1 and TOMM40 genes). SNPs=single nucleotide polymorphisms; HDL=high density lipoprotein; LDL=low density lipoprotein

Using information on all genetic variants associated with the cardiometabolic factors and C reactive protein, we observed that BMI, high density lipoprotein cholesterol, and C reactive protein were inversely associated with Alzheimer’s disease, whereas low density lipoprotein cholesterol and total cholesterol were positively associated (fig I in appendix 2). A genetic variant near the APOE gene, however, was associated with these risk factors and strongly associated with Alzheimer’s disease (P<5×10^−464^), and none of the associations remained after we excluded the pleiotropic variant (fig 3). Genetically predicted waist to hip ratio adjusted for BMI, type 2 diabetes, fasting glucose, fasting insulin, systolic and diastolic blood pressure, and triglycerides were not associated with AD (fig 3). The lack of association with systolic blood pressure remained in sensitivity analyses that excluded a genetic variant (rs7107356) that was strongly associated with AD (odds ratio 0.88, 95% confidence interval 0.72 to 1.07; P=0.20), after we excluded 11 genetic variants associated with AD at P<0.05 (0.92, 0.74 to 1.13; P=0.41), and when we restricted the analysis to the 50 variants with the strongest association with systolic blood pressure (1.03, 0.80 to 1.33; P=0.84).

## Discussion

With genetic variants as proxies for the modifiable risk factors, this Mendelian randomisation analysis supports the evidence from conventional analyses that higher educational attainment is associated with reduced risk of Alzheimer’s disease. We also found suggestive evidence for an inverse association between genetically predicted intelligence and risk. There was also suggestive evidence for possible associations of genetically predicted quantity of smoking, coffee consumption, and 25(OH)D concentrations, but the associations with smoking and coffee were in opposite direction to those observed in conventional analyses (table A in appendix 1). There was no evidence to support associations with alcohol consumption, serum folate, serum vitamin B_12_, total homocysteine, cardiometabolic factors, and C reactive protein.

### Strengths and limitations of study

Strengths of this study include the assessment of multiple potentially modifiable risk factors in relation to Alzheimer’s disease, the use of data from large genome-wide association studies of the risk factors, and the Mendelian randomisation design. This design technique avoids bias from reverse causation and generally reduces confounding by other modifiable environmental exposures. Inference of causality in such analyses, however, relies on the assumptions that the genetic variants used as instruments are strongly associated with the risk factor (assumption 1 in fig 1) and that a pleiotropic or direct causal pathway does not explain the association (assumptions 2 and 3 in fig 1). We cannot exclude that our findings might have been affected by weak instrument bias, which depends on the strength of the genetic instrument through the F statistic.^39^ Instrument strength was low for years of education completed but was considered to be adequate for intelligence and the other risk factors (table B in appendix 1). As the investigations were undertaken in a two sample setting (in which genetic associations with the risk factor and with the disease were estimated in separate datasets), however, any bias from weak instruments is in the direction of the null.^40^ Thus, weak instrument bias cannot explain the observed association between educational attainment and Alzheimer’s. Completely ruling out pleiotropy (where a genetic variant is associated with more than one risk factor) or an alternative direct causal pathway is a challenge for all Mendelian randomisation analyses, particularly for risk factors determined by multiple genetic variants. In this study, we applied four methods: the conventional inverse variance weighted, weighted median, penalised weighted median, and MR-Egger methods. The weighted median approaches give more weight to more precise instrumental variables and the estimate is consistent even when up to 50% of the information comes from invalid or weak instruments.^38^ Results were similar in the inverse variance weighted and the two weighted median analyses. A limitation is that the estimates from the MR-Egger method were imprecise, in particular for completion of college/university education and intelligence. As a consequence, the MR-Egger method could not reliably detect either pleiotropic or causal effects. Another potential source of bias in Mendelian randomisation analyses is population stratification. Nevertheless, this was reduced in our study because the IGAP dataset was restricted to individuals of European ancestry. A further weakness is that power was limited for some of the analyses, and therefore we cannot exclude type II error as an explanation for the null results.

Another potential limitation is that the studies participating in IGAP used somewhat different diagnostic criteria for Alzheimer’s disease, but all cases met standard criteria for possible, probable, or definite Alzheimer’s (appendix 3). Some misclassification, however, was inevitable. A clinical diagnosis with standard criteria has good sensitivity and specificity for discerning between people with and without dementia, but the ability to separate Alzheimer’s from other causes of dementia is less accurate.^1^

### Comparison with other studies

Among potentially modifiable risk factors, the evidence from conventional observational studies consistently supports the association between educational attainment and Alzheimer’s disease.^5^ A previous Mendelian randomisation analysis found no evidence of an association between educational attainment and Alzheimer’s, but the analysis was based on a single genetic variant for length of education and only two variants for university completion.^41^ In our analysis, with data from genome-wide association studies with up to about 405 000 individuals,^21^ length of education conferred by 152 genetic variants and completion of college/university education conferred by 32 variants were significantly associated with Alzheimer’s. Moreover, genebased genome-wide analyses have shown that educational attainment is strongly genetically correlated with intelligence (*r*_g_=0.70), cognitive performance (*r*_g_=0.75), and Alzheimer’s (*r*_g_=−0.31-−0.36).^18^ ^21^

Our findings corroborate the results from previous Mendelian randomisation analyses showing no associations of genetically predicted BMI (based on 32 single nucleotide polymorphisms),^41^ ^42^ ^43^ diabetes,^41^ fasting glucose and insulin (based on 10 single nucleotide polymorphisms),^41^ cholesterol (with exclusion of genetic variants near APOE),^41^ ^44^ ^45^ ^46^ or triglycerides^41^ ^44^ with Alzheimer’s disease. We also found no evidence of an association between abdominal obesity (waist to hip ratio adjusted for BMI) and Alzheimer’s. These null findings suggest that the associations between metabolic factors and hypercholesterolaemia and risk observed in some conventional observational studies^5^ ^9^ could reflect reverse causation bias or confounding—for example, by APOE, which has numerous roles in pathogenesis of Alzheimer’s.^47^

We found no association between systolic blood pressure and Alzheimer’s disease when we used about 100 genetic variants or when we restricted the analysis to the 50 single nucleotide polymorphisms that were most strongly associated with systolic blood pressure. This contrasts with an earlier Mendelian randomisation analysis,^41^ which showed an inverse association based on 24 variants. There are several possible explanations for this disparity. One explanation is that the earlier finding was a false positive and that the present analysis, in which the genetic variants associated with systolic blood pressure explain a larger proportion of variance, shows the true null association. Another explanation is that with a larger number of variants, the potential for pleiotropy is greater, which could have diluted the association in our analysis. A further complicating factor is survival bias as individuals with a high burden of variants associated with systolic blood pressure might have higher mortality and therefore be less represented among people with Alzheimer’s. Randomised controlled trials investigating the effect of antihypertensive treatment on all cause dementia have been inconclusive, and no effect on incidence of Alzheimer’s specifically has been observed.^14^

### Interpretation of findings

There are several plausible pathways that could underlie the associations between higher educational attainment and intelligence and lower risk of Alzheimer’s disease (fig J in appendix 2). One pathway is through increased cognitive reserve, which refers to the ability to recruit alternative brain networks or cognitive paradigms or to use brain structures or networks not normally used to compensate for brain ageing.^19^ ^20^ This implies that an individual with more cognitive reserve (for instance from higher education or intelligence) uses more efficient processing pathways and can sustain more Alzheimer’s pathology before the initial clinical signs and symptoms emerge compared with an individual with less cognitive reserve.^20^

The association between education and Alzheimer’s might also be mediated by health behaviours and downstream metabolic and nutritional factors (fig J in appendix 2). Genetically predicted education was associated with smoking, high density lipoprotein cholesterol, triglycerides, insulin, and BMI (table D in appendix 1). These modifiable factors, however, were not significantly associated with Alzheimer’s and therefore are not likely to be mediators or confounders of the association with education. We were unable to use Mendelian randomisation to examine associations with physical activity and healthy eating patterns, which have been found to be associated with lower risk of Alzheimer’s in conventional observational studies (table A in appendix 1).^5^ Educational attainment could also be associated with occupation, and hence potential exposure to occupational hazards, as well as medication use, depression, and chronic stress, which could influence the risk (fig J in appendix 2). Evidence indicates that certain antidepressants (such as selective serotonin reuptake inhibitors) could stimulate neurogenesis in the hippocampus under certain conditions, while prolonged stress might result in hippocampal atrophy,^48^ which is a modest predictor of progression of mild cognitive impairment to Alzheimer’s.^49^

Shared biological processes that impact on educational attainment and intelligence as well as development of Alzheimer’s might explain some of the associations. The genetic variants associated with educational attainment and intelligence are largely found in genes expressed in brain tissue and are enriched for biological pathways involved in neural and cell development.^18^ ^21^

### Conclusions and future research

Using a genetic approach, we found evidence that higher educational attainment is associated with a reduced risk of Alzheimer’s disease. Our study also provides suggestive evidence that the correlated trait of intelligence is inversely associated with Alzheimer’s. Further research is necessary to understand the pathways underpinning these associations. Furthermore, more work is needed to determine the possible role of smoking, coffee consumption, and vitamin D.

**What is already known on this topic**

Conventional observational studies have shown that educational attainment is associated with the risk of Alzheimer’s disease
Evidence for the associations between lifestyle behaviours and cardiometabolic factors and risk of Alzheimer’s disease is inconclusive
Available data on modifiable risk factors in relation to Alzheimer’s disease are primarily from conventional observational studies, which are vulnerable to confounding and reverse causation bias

**What this study adds**

A Mendelian randomisation approach shows that a genetic predisposition towards longer education is associated with lower odds of Alzheimer’s disease
This study found suggestive evidence of possible associations between higher intelligence, smoking, and concentrations of 25-hydroxyvitamin D and lower odds of Alzheimer’s disease and between higher coffee consumption and higher odds of Alzheimer’s disease

## Web Extra

Extra material supplied by the author

Appendix 2: Supplementary figures A-J

Appendix 3: Description of the original genetic analyses and diagnostic assessment

Appendix 4: Further details of contribution from the International Genomics of Alzheimer’s Project

## Notes

Contributors: SCL analysed the data, drew the figures, and wrote the first draft of the manuscript. All authors contributed to the interpretation of the results and critical revision of the manuscript for important intellectual content and approved the final version of the manuscript. For the International Genomics of Alzheimer’s Project the investigators contributed to the design and implementation of this specific study and/or provided data but did not participate in analysis or writing of the present study. See appendix 4 for further information. SCL is guarantor.

Ethical approval: This study is based on publicly available summarised data. Individual studies within each genome-wide association study had received approval from a relevant institutional review board, and informed consent was obtained from participants or from a caregiver, legal guardian, or other proxy.

Data sharing: No additional data available.

Transparency: The lead author (SCL) affirms that the manuscript is an honest, accurate, and transparent account of the study being reported; that no important aspects of the study have been omitted; that discrepancies from the study as planned have been explained, and that the paper conforms to transparency policy of the International Committee of Medical Journal Editors uniform requirement for manuscripts submitted to biomedical journals.
