# Supplementary material for: Modifiable pathways in Alzheimer’s disease: Mendelian randomisation analysis
Source: BMJ. 2017 Dec 7;359:j5375. doi: 10.1136/bmj.j5375 (PMC5717765; doi:10.1136/bmj.j5375)
Supplement: Supplementary file 1 — Appendix 1: Supplementary tables A-D [file lars039122.ww1.pdf]

Appendix 1: Supplementary tables [posted as supplied by author]

Table A. Summary results from meta-analyses of prospective observational studies of potentially modifiable risk factors, including socioeconomic, lifestyle and dietary, cardiometabolic, and inflammatory factors, in relation to Alzheimer's disease

Potentially modifiable risk factor	Direction of association	Relative risk (95% CI)	I^2	Studies	Reference	Included in MR analysis	Reason for not including in MR*
**Socioeconomic factors**							
Educational attainment (high versus low)	↓	0.80 (0.72 to 0.89)	93%	20	Xu et al, 2015[1]	√	
Educational attainment (low versus high)	↑	1.35 (1.12 to 1.58)	57%	16	Xu et al, 2015[1]	√	
Socioeconomic status (low versus high)†	↑	2.55 (1.55 to 3.55)	0%	4	Xu et al, 2015[1]		1
**Lifestyle and dietary factors**							
Smoking (current versus non-smoking)	↑	1.44 (1.11 to 1.76)	77%	14	Xu et al, 2015[1]	√	
Alcohol (ever versus never consumption)	↓	0.43 (0.17 to 0.69)	7%	3	Xu et al, 2015[1]	√	
Alcohol (moderate drinking 1-3 drinks/day)	↓	0.61 (0.54 to 0.68)	0%	5	Xu et al, 2015[1]	√	
Coffee (high versus low consumption)	↓	0.73 (0.55 to 0.97)	0%	4	Liu et al, 2015[2]	√	
Physical activity, leisure-time (high versus low)	↓	0.67 (0.52 to 0.81)	81%	13	Xu et al, 2015[1]		1
25-hydroxyvitamin D (<50 nmol/L versus above)	↑	1.21 (1.01 to 1.40)	0%	2§	Shen and Ji, 2015[3]	√	
Folate levels (low versus high)	↑	1.41 (1.07 to 1.75)	0%	3	Xu et al, 2015[1]	√	
Folate levels (high versus low)	↓	0.93 (0.88 to 0.98)	13%	2	Xu et al, 2015[1]	√	
Holotranscobalamin (high versus low)	↓	0.98 (0.97 to 1.00)	0%	2	Xu et al, 2015[1]	√¶	
Vitamin B_{12} (high versus low)	NA	0.93 (0.68 to 1.17)	0%	2	Xu et al, 2015[1]	√¶	
Total homocysteine levels (high versus low)	↑	1.15 (1.02 to 1.27)	45%	8	Xu et al, 2015[1]	√	
Vitamin C intake (high versus low)	↓	0.74 (0.55 to 0.93)	0%	6	Xu et al, 2015[1]		1
Vitamin E intake (high versus low)	↓	0.73 (0.62 to 0.84)	0%	6	Xu et al, 2015[1]		1
Eicosapentaenoic acid (high versus low)	NA	0.96 (0.75 to 1.16)	0%	3	Xu et al, 2015[1]		2
Docosahexaenoic acid (high versus low)	NA	0.70 (0.37 to 1.03)	68%	4	Xu et al, 2015[1]		2
Total omega-3 fatty acids (high versus low)	NA	0.81 (0.39 to 1.23)	70%	3	Xu et al, 2015[1]		2
Fish consumption (high versus low)	↓	0.66 (0.43 to 0.90)	60%	6	Xu et al, 2015[1]		1
Mediterranean-type dietary pattern	↓	0.56 (0.41 to 0.71)	46%	4	Xu et al, 2015[1]		1

**Cardiometabolic factors**							
Obesity (BMI ≥30 kg/m^2) in midlife	↑	1.61 (1.11 to 2.12)	69%	6	Xu et al, 2015[1]	√	
Obesity (BMI ≥30 kg/m^2) in late-life	↓	0.80 (0.64 to 0.97)	73%	9	Xu et al, 2015[1]	√	
Diabetes in midlife	↑	1.40 (1.25 to 1.57)	11%	4	Meng et al, 2014[4]	√	
Diabetes in midlife and late-life	↑	1.33 (1.14 to 1.52)	70%	23	Xu et al, 2015[1]	√	
Fasting insulin levels (high versus low)	NA	1.19 (0.96 to 1.42)	0%	3	Xu et al, 2015[1]	√	
Systolic blood pressure (≥160 mmHg)	↑	1.04 (1.01 to 1.08)	0%	5	Xu et al, 2015[1]	√	
Systolic blood pressure (high versus low) in midlife	NA	1.77 (0.93 to 3.37)	0%	3	Meng et al, 2014[4]	√	
Diastolic blood pressure (high versus low) in midlife	↑	2.38 (1.34 to 4.23)	0%	3	Meng et al, 2014[4]	√	
Hypercholesterolemia in midlife	↑	1.72 (1.32 to 2.24)	9%	4	Meng et al, 2014[4]	√	
Hypercholesterolemia in midlife and late-life	NA	0.94 (0.78 to 1.09)	86%	16	Xu et al, 2015[1]	√	
Metabolic syndrome**	↓	0.71 (0.49 to 0.93)	37%	3	Xu et al, 2015[1]	√**	
**Inflammatory markers**							
C-reactive protein levels (high versus low)	↑	1.21 (1.03 to 1.42)	0%	6	Koyama et al, 2013[5]	√	
Interleukin 6 levels (high versus low)	NA	1.06 (0.83 to 1.35)	0%	5	Koyama et al, 2013[5]		2

BMI = body mass index; CI = confidence interval; MR = Mendelian randomisation; NA = no association. *Reasons for not assessing the association between the modifiable factor and Alzheimer's disease using the Mendelian randomisation approach were: 1) no genome-wide significant ($P<5\times10^{-8}$) genetic variants associated with the risk factor were identified; 2) no evidence of an association between the modifiable factor and Alzheimer's disease. †Combination of education and income or education and occupation. ‡Inverse association in meta-analysis of 9 case-control studies (0.60; 0.37 to 0.84).[1] §Two prospective studies have been published since this meta-analysis of which one showed an increased risk of Alzheimer's disease associated with vitamin D deficiency (2.85; 1.37 to 5.97),[6] whereas the other study found no association.[7] ¶Holotranscobalamin is as the active form of vitamin B_{12} (and may be a more sensitive marker of vitamin B_{12} deficiency). Despite of the lack of overall association of vitamin B_{12} with Alzheimer's disease, serum vitamin B_{12} was included in the Mendelian randomisation analysis because it is one of the main determinants of homocysteine levels. **Components of the metabolic syndrome include abdominal obesity, high triglyceride levels, low high-density lipoprotein cholesterol levels, hypertension, and elevated fasting glucose levels. Each component of the metabolic syndrome (abdominal obesity [waist-to-hip ratio], triglycerides, high-density lipoprotein cholesterol, systolic and diastolic blood pressure, and fasting glucose) was evaluated for an association with Alzheimer's disease in the present Mendelian randomisation analysis.

Table B. Genome-wide association studies and number of single nucleotide polymorphisms used as instrumental variable in the Mendelian randomisation analyses of potentially modifiable risk factors in relation to Alzheimer's disease

	Published genome-wide association study of the modifiable risk factor				Present Mendelian randomisation analysis			
**Modifiable risk factor**	**Reference**	**Maximum sample size**	**No. of independent genome-wide significant SNPs***	**R^2 (%) explained by the SNPs**	**No. of SNPs included in the analysis**	**F statistic (instrument al strength)**	**Proxies used (r^2 >0.9)**	**Excluded (no proxy at r^2 >0.9)**
**Education and intelligence**								
Years of education	Okbay et al, 2016[8]	405 072	162	1.6	152	5.7	10	10
College/university	Okbay et al, 2016[8]	280 007	34	NA	32	NA	4	2
Intelligence	Sniekers et al, 2017[9]	78 307	18	2.0-4.8	16	60-144		2
**Lifestyle/dietary factors**								
Smoking (cigarettes per day)	Thorgeirsson et al, 2010[10]	86 956	4	0.5	4	68		
Smoking initiation (ever versus never smoker)	TGC, 2010[11]	143 023	1	0.03	1	16		
Smoking cessation (former versus current)	TGC, 2010[11]	64 924	1	0.19	1	103		
Alcohol consumption	Jorgenson et al, 2017[12]	71 071	3†	NA	2	NA		
Coffee consumption	Cornelis et al, 2015[13]	129 488	5	1.3	5	140		
25-hydroxyvitamin D	Vimaleswaran et al, 2013[14]	42 024	4	3.6	4	487		
Serum folate	Grarup et al, 2017[15]	37 456	2	1.0	2	271		
Serum vitamin B12	Grarup et al, 2017[15]	45 576	11	6.3	7	310		
Total homocysteine	van Meurs et al, 2013[16]	44 147	18	5.9	18	178		
**Cardiometabolic factors**								
Body mass index	Locke et al, 2015[17]	322 154	77‡	2.4‡	77	17‡		
Waist-to-hip ratio adjusted for body mass index	Shungin et al 2015[18]	210 023	39§	1.2§	38	17§		1
Type 2 diabetes	Morris et al, 2012[19]	149 821	50¶	5.7	50	62		
Fasting glucose	Scott et al, 2012[20]	133 010	36	4.8	36	72		
Fasting insulin	Scott et al, 2012[20]	133 010	19	1.2	19	34		
Systolic blood pressure	Hoffmann et al, 2017[21]	321 262	103	2.9	93	15	6	10
Diastolic blood pressure	Hoffmann et al, 2017[21]	321 262	118	2.5	105	11	7	13

HDL cholesterol	GLGC, 2013[22]	188 577	71	13.7	71	105		
LDL cholesterol	GLGC, 2013[22]	188 577	57	14.6	57	139		1
Total cholesterol	GLGC, 2013[22]	188 577	74	15	74	110		1
Triglycerides	GLGC, 2013[22]	188 577	40	11.7	40	158	1	
**Inflammatory markers**								
C-reactive protein	Dehghan et al, 2011[23]	82 725	18	4.9	18	147	2	

GLGC = Global Lipids Genetics Consortium; HDL = high-density lipoprotein; LDL = low-density lipoprotein; SNP = single nucleotide polymorphism. *Number of independent genome-wide significant ($P<5\times10^{-8}$) SNPs identified in the sex-combined meta-analysis of discovery and replication samples. Independent was defined as not in linkage disequilibrium ($r^2<0.2$) with other SNPs for the same risk factor. †Including two SNPs nearby the *KLB* and *GCKR* genes that were genome-wide significant in the trans-ethnic meta-analysis and one SNP in the *ADH1B* that was highly significant in non-Hispanic whites but not in the combined trans-ethnic sample. ‡SNPs from the primary sex-combined genome-wide meta-analysis of European-descent individuals; twenty genome-wide significant SNPs identified in subgroup-analysis of only women or men or trans-ethnic analysis were not included. Variance and F statistic are estimated on the basis of the 77 SNPs. §SNPs from the primary sex-combined genome-wide meta-analysis of European-descent individuals; eight SNPs that were genome-wide significant in sex-specific analysis only were omitted. Variance and F statistic are estimated on the basis of the 39 SNPs. ¶The 50 SNPs represent the lead SNP for each genome-wide significant loci associated with diabetes of which 39 SNPs were genome-wide significant in the combined meta-analysis by Morris et al, 2012[19] and 11 were significant in earlier genome-wide association studies.[24-26] Summary statistics from Morris et al, 2012[19] were used for all SNPs.

Table C. Summary statistics for the genetic variants associated with the modifiable risk factors investigated for an association with Alzheimer's disease in the present Mendelian randomisation study

					Risk factor		Alzheimer's disease results*		
Risk factor	SNP	Chr	Nearest gene	EA	β†	SE	β	SE	P value
Education	rs1008078	1	NA	C	0.017	0.002	-0.0364	0.0163	0.0258
Education	rs11588857	1	NA	A	0.020	0.003	0.0085	0.0186	0.6480
Education	rs12076635	1	NA	C	0.018	0.003	0.0124	0.0192	0.5202
Education	rs12134151	1	NA	G	0.013	0.002	0.0099	0.0157	0.5270
Education	rs12142680	1	NA	A	0.026	0.004	-0.0214	0.0597	0.7203
Education	rs12143094	1	NA	C	0.029	0.005	-0.1470	0.0833	0.0775
Education	rs12145291	1	NA	C	0.029	0.005	-0.0491	0.0340	0.1487
Education	rs12410444	1	NA	G	0.017	0.002	-0.0124	0.0171	0.4705
Education	rs12754946	1	NA	T	0.013	0.002	-0.0155	0.0166	0.3505
Education	rs17372140	1	NA	G	0.014	0.002	-0.0162	0.0174	0.3498
Education	rs1766964	1	NA	T	0.022	0.004	-0.0227	0.0262	0.3865
Education	rs2568955	1	NA	C	0.016	0.003	-0.0241	0.0193	0.2124
Education	rs2992632	1	NA	A	0.016	0.002	-0.0468	0.0178	0.0086
Education	rs301800	1	NA	T	0.016	0.003	-0.0052	0.0195	0.7891
Education	rs34305371	1	NA	A	0.036	0.004	-0.0531	0.0356	0.1362
Education	rs35771425	1	NA	T	0.018	0.003	-0.0007	0.0187	0.9693
Education	rs4378243	1	NA	T	0.018	0.003	-0.0056	0.0219	0.8000
Education	rs56044892	1	NA	C	0.016	0.003	-0.0076	0.0230	0.7425
Education	rs648163	1	NA	T	0.014	0.003	-0.0007	0.0178	0.9693
Education	rs78365243	1	NA	T	0.029	0.005	0.0214	0.0376	0.5695
Education	rs10178115	2	NA	T	0.014	0.002	-0.0070	0.0158	0.6597
Education	rs10930008	2	NA	G	0.014	0.003	-0.0151	0.0188	0.4230
Education	rs11687170	2	NA	T	0.021	0.003	-0.0321	0.0214	0.1340
Education	rs12694681	2	NA	T	0.014	0.002	0.0335	0.0176	0.0570
Education	rs12987662	2	NA	A	0.025	0.002	-0.0238	0.0159	0.1346
Education	rs1596747	2	NA	A	0.014	0.002	-0.0289	0.0158	0.0671
Education	rs1606974	2	NA	A	0.022	0.003	-0.0226	0.0259	0.3840
Education	rs16845580	2	NA	T	0.016	0.002	-0.0266	0.0160	0.0973
Education	rs17504614	2	NA	T	0.016	0.003	-0.0629	0.0210	0.0028
Education	rs17824247	2	NA	C	0.016	0.002	0.0031	0.0158	0.8436
Education	rs268134	2	NA	A	0.014	0.003	0.0482	0.0178	0.0066
Education	rs34106693	2	NA	C	0.017	0.003	-0.0062	0.0233	0.7897
Education	rs356992	2	NA	C	0.017	0.002	-0.0369	0.0173	0.0334
Education	rs4500960	2	NA	C	0.014	0.002	-0.0233	0.0160	0.1454
Education	rs4675248	2	NA	G	0.012	0.002	-0.0058	0.0159	0.7167
Education	rs4851251	2	NA	C	0.015	0.002	-0.0251	0.0182	0.1668
Education	rs56158183	2	NA	A	0.025	0.004	-0.0595	0.0350	0.0893
Education	rs6715849	2	NA	G	0.015	0.002	0.0175	0.0156	0.2616
Education	rs71413877	2	NA	A	0.035	0.005	-0.0035	0.0921	0.9697
Education	rs7590368	2	NA	C	0.014	0.003	-0.0007	0.0173	0.9685
Education	rs7593947	2	NA	A	0.015	0.002	-0.0368	0.0163	0.0236

Education	rs76076331	2	NA	T	0.020	0.003	0.0054	0.0240	0.8230
Education	rs77702819	2	NA	T	0.022	0.004	0.0039	0.0305	0.8973
Education	rs11130222	3	NA	A	0.025	0.002	0.0129	0.0157	0.4097
Education	rs113011189	3	NA	C	0.025	0.004	-0.0513	0.0618	0.4063
Education	rs13090388	3	NA	T	0.026	0.002	-0.0082	0.0174	0.6394
Education	rs2624818	3	NA	A	0.021	0.004	-0.0570	0.0266	0.0319
Education	rs3172494	3	NA	T	0.023	0.003	0.0642	0.0253	0.0113
Education	rs34638686	3	NA	T	0.023	0.004	-0.0259	0.0257	0.3142
Education	rs55786114	3	NA	C	0.030	0.004	0.0393	0.0387	0.3105
Education	rs56262138	3	NA	A	0.014	0.002	0.0029	0.0194	0.8828
Education	rs62263033	3	NA	T	0.037	0.006	-0.1114	0.0796	0.1619
Education	rs62263923	3	NA	G	0.017	0.002	0.0057	0.0161	0.7243
Education	rs6806509	3	NA	T	0.018	0.003	0.0173	0.0218	0.4271
Education	rs71326918	3	NA	A	0.022	0.004	0.0021	0.0384	0.9562
Education	rs7429990	3	NA	C	0.015	0.003	0.0349	0.0176	0.0473
Education	rs7610856	3	NA	A	0.012	0.002	0.0059	0.0157	0.7081
Education	rs7633857	3	NA	G	0.014	0.002	0.0183	0.0156	0.2404
Education	rs9755467	3	NA	T	0.019	0.003	-0.0243	0.0205	0.2367
Education	rs12640626	4	NA	A	0.013	0.002	0.0140	0.0158	0.3740
Education	rs1912528	4	NA	T	0.014	0.002	-0.0041	0.0161	0.8005
Education	rs1967109	4	NA	G	0.017	0.003	-0.0051	0.0215	0.8125
Education	rs4308415	4	NA	G	0.013	0.002	-0.0049	0.0155	0.7513
Education	rs4863692	4	NA	T	0.017	0.002	0.0085	0.0168	0.6132
Education	rs6839705	4	NA	A	0.015	0.002	-0.0015	0.0161	0.9245
Education	rs10223052	5	NA	A	0.016	0.002	-0.0217	0.0164	0.1856
Education	rs113474297	5	NA	C	0.021	0.003	-0.0478	0.0231	0.0380
Education	rs12653396	5	NA	T	0.013	0.002	0.0006	0.0155	0.9716
Education	rs146723842	5	NA	C	0.014	0.002	0.0040	0.0171	0.8144
Education	rs152603	5	NA	G	0.013	0.002	0.0013	0.0166	0.9385
Education	rs1562242	5	NA	C	0.013	0.002	-0.0079	0.0156	0.6108
Education	rs4493682	5	NA	C	0.019	0.003	-0.0041	0.0200	0.8389
Education	rs61160187	5	NA	G	0.017	0.002	-0.0267	0.0163	0.1007
Education	rs62379838	5	NA	T	0.013	0.002	0.0010	0.0172	0.9554
Education	rs660001	5	NA	G	0.018	0.003	0.0022	0.0194	0.9102
Education	rs6882046	5	NA	G	0.019	0.003	-0.0436	0.0170	0.0103
Education	rs700590	5	NA	C	0.019	0.002	-0.0175	0.0158	0.2695
Education	rs11756123	6	NA	T	0.015	0.002	-0.0395	0.0165	0.0163
Education	rs1338554	6	NA	A	0.015	0.002	-0.0142	0.0157	0.3650
Education	rs2179152	6	NA	C	0.013	0.002	-0.0213	0.0167	0.2035
Education	rs56081191	6	NA	A	0.028	0.004	0.0012	0.0368	0.9750
Education	rs56231335	6	NA	C	0.017	0.002	-0.0471	0.0187	0.0117
Education	rs6939294	6	NA	T	0.016	0.003	-0.0282	0.0177	0.1113
Education	rs7772172	6	NA	A	0.013	0.002	0.0099	0.0156	0.5251
Education	rs9349957	6	NA	T	0.021	0.003	0.0302	0.0251	0.2291
Education	rs9401593	6	NA	C	0.024	0.002	-0.0231	0.0156	0.1393
Education	rs10215082	7	NA	G	0.014	0.002	0.0248	0.0167	0.1364
Education	rs113520408	7	NA	A	0.015	0.002	0.0148	0.0175	0.3991
Education	rs113779084	7	NA	A	0.014	0.002	0.0036	0.0172	0.8331

Education	rs11771168	7	NA	C	0.015	0.003	0.0171	0.0190	0.3685
Education	rs11771419	7	NA	A	0.016	0.003	0.0033	0.0196	0.8676
Education	rs11976020	7	NA	G	0.015	0.003	0.0028	0.0195	0.8859
Education	rs12531458	7	NA	A	0.012	0.002	0.0004	0.0155	0.9783
Education	rs12702087	7	NA	A	0.013	0.002	0.0109	0.0155	0.4819
Education	rs17167170	7	NA	A	0.019	0.003	-0.0244	0.0197	0.2156
Education	rs2944833	7	NA	G	0.014	0.002	-0.0112	0.0158	0.4798
Education	rs320700	7	NA	A	0.014	0.002	-0.0050	0.0162	0.7554
Education	rs7791133	7	NA	C	0.014	0.002	0.0008	0.0161	0.9620
Education	rs11774212	8	NA	T	0.016	0.002	-0.0157	0.0174	0.3658
Education	rs11782074	8	NA	G	0.016	0.002	0.0260	0.0164	0.1141
Education	rs10818606	9	NA	C	0.014	0.002	-0.0085	0.0161	0.5963
Education	rs10821136	9	NA	T	0.013	0.002	-0.0149	0.0163	0.3607
Education	rs11998763	9	NA	A	0.017	0.002	-0.0065	0.0157	0.6792
Education	rs17425572	9	NA	A	0.014	0.002	-0.0039	0.0156	0.8027
Education	rs4741343	9	NA	G	0.016	0.003	0.0011	0.0206	0.9558
Education	rs4741351	9	NA	G	0.015	0.002	-0.0092	0.0182	0.6145
Education	rs7029201	9	NA	A	0.025	0.002	-0.0241	0.0172	0.1602
Education	rs7033137	9	NA	C	0.015	0.003	0.0063	0.0203	0.7560
Education	rs10761741	10	NA	T	0.013	0.002	-0.0221	0.0156	0.1561
Education	rs10786662	10	NA	G	0.017	0.002	0.0157	0.0155	0.3125
Education	rs12761761	10	NA	T	0.016	0.003	-0.0104	0.0194	0.5908
Education	rs149613931	10	NA	G	0.028	0.005	-0.0538	0.0341	0.1145
Education	rs1925576	10	NA	G	0.012	0.002	-0.0347	0.0155	0.0250
Education	rs61874768	10	NA	G	0.016	0.003	-0.0049	0.0209	0.8146
Education	rs73344830	10	NA	A	0.015	0.002	0.0043	0.0161	0.7884
Education	rs7914680	10	NA	G	0.014	0.002	0.0071	0.0170	0.6768
Education	rs10444359	11	NA	G	0.014	0.003	-0.0045	0.0188	0.8101
Education	rs111321694	11	NA	C	0.016	0.003	0.0036	0.0239	0.8796
Education	rs1550973	11	NA	G	0.014	0.002	-0.0141	0.0171	0.4072
Education	rs7945718	11	NA	A	0.014	0.002	-0.0583	0.0169	0.0006
Education	rs7948975	11	NA	T	0.014	0.002	0.0349	0.0183	0.0567
Education	rs79925071	11	NA	T	0.013	0.002	0.0142	0.0178	0.4256
Education	rs10772644	12	NA	C	0.020	0.003	0.0048	0.0255	0.8508
Education	rs10773002	12	NA	A	0.022	0.003	-0.0454	0.0177	0.0105
Education	rs1389473	12	NA	G	0.013	0.002	-0.0136	0.0166	0.4126
Education	rs2456973	12	NA	C	0.019	0.002	-0.0150	0.0164	0.3620
Education	rs7964899	12	NA	A	0.016	0.002	-0.0162	0.0156	0.2967
Education	rs8002014	13	NA	G	0.024	0.003	0.0034	0.0185	0.8536
Education	rs9556958	13	NA	C	0.015	0.002	0.0250	0.0158	0.1142
Education	rs10483349	14	NA	G	0.017	0.003	-0.0258	0.0199	0.1953
Education	rs1115240	14	NA	G	0.016	0.003	-0.0120	0.0177	0.4998
Education	rs34344888	14	NA	G	0.016	0.002	-0.0002	0.0159	0.9921
Education	rs58694847	14	NA	G	0.017	0.003	0.0125	0.0177	0.4799
Education	rs12900061	15	NA	A	0.019	0.003	-0.0069	0.0207	0.7383
Education	rs1378214	15	NA	C	0.015	0.002	-0.0242	0.0159	0.1288
Education	rs281302	15	NA	G	0.013	0.002	-0.0108	0.0161	0.5023
Education	rs28420834	15	NA	G	0.014	0.002	-0.0296	0.0168	0.0784

Education	rs4076457	15	NA	T	0.015	0.003	-0.0055	0.0185	0.7645
Education	rs6493271	15	NA	T	0.017	0.003	0.0125	0.0204	0.5397
Education	rs11643654	16	NA	A	0.013	0.002	-0.0300	0.0174	0.0841
Education	rs8049439	16	NA	T	0.015	0.002	0.0196	0.0163	0.2298
Education	rs9914544	17	NA	C	0.013	0.002	0.0154	0.0159	0.3341
Education	rs12956009	18	NA	C	0.013	0.002	0.0058	0.0156	0.7077
Education	rs62100765	18	NA	C	0.015	0.002	0.0263	0.0158	0.0964
Education	rs7241530	18	NA	C	0.013	0.002	-0.0161	0.0192	0.4027
Education	rs9964724	18	NA	T	0.018	0.002	-0.0023	0.0171	0.8930
Education	rs115945269	19	NA	C	0.029	0.005	-0.0087	0.1139	0.9393
Education	rs12462428	19	NA	T	0.016	0.003	-0.0002	0.0201	0.9917
Education	rs1382358	19	NA	T	0.020	0.003	-0.0336	0.0283	0.2353
Education	rs6065080	20	NA	C	0.013	0.002	0.0116	0.0167	0.4870
Education	rs35532491	22	NA	T	0.022	0.004	0.0146	0.0258	0.5721
Education	rs7286601	22	NA	G	0.014	0.002	0.0080	0.0188	0.6713
College/university	rs1008078	1	NA	C	0.035	0.003	-0.0364	0.0163	0.0258
College/university	rs11588857	1	NA	A	0.042	0.003	0.0085	0.0186	0.6480
College/university	rs34305371	1	NA	A	0.073	0.005	-0.0531	0.0356	0.1362
College/university	rs72943001	1	NA	C	0.042	0.003	-0.0152	0.0204	0.4544
College/university	rs2309757	2	NA	C	0.054	0.003	-0.0224	0.0160	0.1608
College/university	rs2457660	2	NA	C	0.035	0.003	-0.0346	0.0175	0.0484
College/university	rs7597412	2	NA	C	0.034	0.003	0.0188	0.0160	0.2399
College/university	rs76076331	2	NA	T	0.046	0.004	0.0054	0.0240	0.8230
College/university	rs11712056	3	NA	T	0.049	0.003	-0.0053	0.0164	0.7475
College/university	rs148734725	3	NA	A	0.044	0.003	-0.0069	0.0177	0.6959
College/university	rs9686024	4	NA	A	0.044	0.004	-0.0151	0.0213	0.4795
College/university	rs10223052	5	NA	A	0.035	0.003	-0.0217	0.0164	0.1856
College/university	rs11241565	5	NA	C	0.033	0.003	0.0023	0.0162	0.8872
College/university	rs61160187	5	NA	G	0.039	0.003	-0.0267	0.0163	0.1007
College/university	rs6882046	5	NA	G	0.043	0.003	-0.0436	0.0170	0.0103
College/university	rs62422661	6	NA	T	0.041	0.003	-0.0322	0.0163	0.0482
College/university	rs9349957	6	NA	T	0.047	0.003	0.0302	0.0251	0.2291
College/university	rs12671937	7	NA	A	0.039	0.003	0.0157	0.0164	0.3382
College/university	rs12699131	7	NA	A	0.033	0.003	-0.0137	0.0160	0.3920
College/university	rs7781266	7	NA	C	0.042	0.003	-0.0130	0.0184	0.4785
College/university	rs7791133	7	NA	C	0.034	0.003	0.0008	0.0161	0.9620
College/university	rs13294439	9	NA	C	0.047	0.003	-0.0239	0.0170	0.1608
College/university	rs12354486	10	NA	C	0.042	0.003	-0.006	0.0208	0.7735
College/university	rs12797960	11	NA	C	0.034	0.003	-0.0425	0.0159	0.0073
College/university	rs1873982	12	NA	A	0.034	0.003	-0.0075	0.0160	0.6402
College/university	rs2456973	12	NA	C	0.037	0.003	-0.015	0.0164	0.3620
College/university	rs7306755	12	NA	A	0.046	0.003	-0.0338	0.0192	0.0778
College/university	rs8002014	13	NA	G	0.049	0.003	0.0034	0.0185	0.8536
College/university	rs58694847	14	NA	G	0.040	0.003	0.0125	0.0177	0.4799
College/university	rs77092939	14	NA	C	0.051	0.004	-0.0370	0.0286	0.1963
College/university	rs117468730	16	NA	G	0.116	0.009	-0.0449	0.0592	0.4491
College/university	rs12957516	18	NA	T	0.037	0.003	0.0193	0.0173	0.2640
Intelligence	rs12744310	1	FOXO6/SCMH1	C	0.0391	0.0067	-0.0126	0.0190	0.5080

Intelligence	rs10191758	2	ARHGAP15	G	0.0370	0.0062	0.0112	0.0159	0.4809
Intelligence	rs13010010	2	LINC01104	T	0.0294	0.0052	-0.0243	0.0165	0.1415
Intelligence	rs6746731	2	ZNF638	G	0.0278	0.0051	-0.0383	0.0156	0.0139
Intelligence	rs6779302	3	DAZL/PLZL2	G	0.0343	0.0063	0.0054	0.0160	0.7341
Intelligence	rs7646501	3	NR1D2/LINC00691	A	0.0378	0.0063	-0.0471	0.0177	0.0079
Intelligence	rs41352752	5	MEF2C	C	0.1012	0.0178	-0.1040	0.0769	0.1762
Intelligence	rs1906252	6	MIR2113	A	0.0334	0.0051	-0.0230	0.0157	0.1439
Intelligence	rs2490272	6	FOXO3	T	0.0389	0.0052	0.0405	0.0163	0.0131
Intelligence	rs10236197	7	PDE1C	T	0.0338	0.0052	-0.0255	0.0164	0.1193
Intelligence	rs4728302	7	EXOC4	C	0.0308	0.0052	-0.0346	0.0157	0.0272
Intelligence	rs11138902	9	APBA1	A	0.0278	0.0051	0.0053	0.0157	0.7373
Intelligence	rs2251499	13	LINC00343/LINC00460	T	0.0363	0.0058	-0.0135	0.0178	0.4462
Intelligence	rs12928404	16	ATXN2L	T	0.0293	0.0051	0.0212	0.0166	0.2023
Intelligence	rs16954078	17	SKAP1	T	0.0375	0.0068	-0.0105	0.0195	0.5896
Intelligence	rs36093924	22	CYP2D7	C	0.0384	0.0061	-0.0206	0.0166	0.2151
Smoking quantity	rs215605	7	NA	G	0.26	0.04	0.0202	0.0165	0.2222
Smoking quantity	rs13280604	8	CHRNB3/CHRNA6	A	0.31	0.05	0.0004	0.0184	0.9830
Smoking quantity	rs1051730	15	CHRNA3/CHRNA5/CHRNB4	A	0.80	0.05	-0.0418	0.0163	0.0103
Smoking quantity	rs4105144	19	CYP2A6/CYP2B6	C	0.39	0.06	-0.0214	0.0348	0.5381
Smoking initiation	rs6265	11	BDNF	C	0.0614	0.011	-0.0215	0.0198	0.2780
Smoking cessation	rs3025343	9	DBH	G	0.1210	0.022	0.0174	0.0267	0.5134
Alcohol	rs4665985	2	GCKR	C	0.04	0.008	0.0003	0.0191	0.9877
Alcohol	rs1229984	4	ADH1B	C	0.19	0.015	-0.0444	0.0424	0.2950
Alcohol	rs7686419	4	KLB	G	0.03	0.008	-0.0383	0.0158	0.0153
Coffee	rs17685	7	POR	A	0.07	0.01	-0.0253	0.0177	0.1515
Coffee	rs4410790	7	AHR	C	0.10	0.01	0.0300	0.0166	0.0706
Coffee	rs7800944	7	MLXIPL	C	0.05	0.01	0.0292	0.0182	0.1087
Coffee	rs2472297	15	CYP1A2	T	0.14	0.01	0.0370	0.0196	0.0592
Coffee	rs9902453	17	EFCAB5	G	0.03	0.01	0.0257	0.0161	0.1110
25OHD	rs2282679	4	GC	T	8.45	0.31	-0.0332	0.0179	0.0627
25OHD	rs10741657	11	CYP2R1	A	3.12	0.29	-0.0110	0.0160	0.4917
25OHD	rs12785878	11	DHCR7	T	3.70	0.30	-0.0223	0.0178	0.2082
25OHD	rs6013897	20	CYP24A1	T	1.85	0.33	-0.0212	0.0195	0.2773
Serum folate	rs1801133	1	MTHFR	G	0.096	NA	-0.0067	0.0163	0.6817
Serum folate	rs652197	11	FOLR3	C	0.069	NA	0.0187	0.0287	0.5154
Vitamin B_{12}	rs2270655	4	MMAA	G	0.066	NA	0.0062	0.0353	0.8616
Vitamin B_{12}	rs1141321	6	MUT	C	0.061	NA	-0.0253	0.0183	0.1671
Vitamin B_{12}	rs1801222	10	CUBN	G	0.110	NA	0.0024	0.0167	0.8838
Vitamin B_{12}	rs34324219	11	TCN1	C	0.210	NA	0.0480	0.0361	0.1843
Vitamin B_{12}	rs3742801	14	ABCD4	T	0.045	NA	0.0102	0.0163	0.5333
Vitamin B_{12}	rs2336573	19	CD320	T	0.320	NA	0.0366	0.0469	0.4343
Vitamin B_{12}	rs1131603	22	TCN2	C	0.190	NA	0.0918	0.0574	0.1098
Homocysteine	rs12134663	1	MTHFR	C	0.1010	0.011	-0.0364	0.0208	0.0806
Homocysteine	rs1801133	1	MTHFR	A	0.1583	0.007	0.0067	0.0163	0.6817
Homocysteine	rs2275565	1	MTR	G	0.0542	0.009	-0.0066	0.0193	0.7336
Homocysteine	rs4660306	1	MMACHC	T	0.0435	0.007	-0.0218	0.0168	0.1951
Homocysteine	rs1047891	2	CPS1	A	0.0864	0.008	-0.0154	0.0195	0.4316
Homocysteine	rs548987	6	SLC17A3	C	0.0597	0.010	-0.0026	0.0243	0.9154

Homocysteine	rs9369898	6	MUT	A	0.0449	0.007	0.0157	0.0157	0.3196
Homocysteine	rs42648	7	GTPB10	G	0.0395	0.007	-0.0062	0.0157	0.6929
Homocysteine	rs12780845	10	CUBN	A	0.0529	0.009	0.0003	0.0165	0.9872
Homocysteine	rs1801222	10	CUBN	A	0.0453	0.007	-0.0024	0.0167	0.8838
Homocysteine	rs7130284	11	NOX4	C	0.1242	0.013	0.0171	0.0303	0.5723
Homocysteine	rs957140	11	NOX4	G	0.0450	0.008	-0.0292	0.0156	0.0606
Homocysteine	rs2251468	12	HNF1A	C	0.0512	0.007	-0.0171	0.0164	0.2973
Homocysteine	rs12921383	16	DPEP1/FANCA	C	0.0900	0.014	0.0472	0.0306	0.1230
Homocysteine	rs154657	16	DPEP1	A	0.0963	0.007	0.0178	0.0169	0.2919
Homocysteine	rs838133	19	FUT2	A	0.0422	0.007	0.0261	0.0167	0.1188
Homocysteine	rs234709	21	CBS	C	0.0718	0.007	-0.0106	0.0158	0.5040
Homocysteine	rs2851391	21	CBS	T	0.0560	0.008	0.0133	0.0160	0.4048
Body mass index	rs11165643	1	PTBP2	T	0.022	0.003	0.0109	0.0158	0.4886
Body mass index	rs11583200	1	ELAVL4	C	0.018	0.003	0.0162	0.0164	0.3239
Body mass index	rs12401738	1	FUBP1	A	0.021	0.003	0.0291	0.0166	0.0787
Body mass index	rs12566985	1	FPGT-TNNI3K	G	0.024	0.003	0.0061	0.0155	0.6957
Body mass index	rs17024393	1	GNAT2	C	0.066	0.009	-0.0168	0.0504	0.7384
Body mass index	rs2820292	1	NAV1	C	0.020	0.003	0.0143	0.0156	0.3611
Body mass index	rs3101336	1	NEGR1	C	0.033	0.003	-0.0146	0.0166	0.3778
Body mass index	rs543874	1	SEC16B	G	0.048	0.004	0.0226	0.0205	0.2696
Body mass index	rs657452	1	AGBL4	A	0.023	0.003	0.0411	0.0164	0.0125
Body mass index	rs10182181	2	ADCY3	G	0.031	0.003	0.0069	0.0154	0.6548
Body mass index	rs11125767	2	LINC01122	C	0.023	0.003	0.0131	0.0173	0.4492
Body mass index	rs11126666	2	KCNK3	A	0.021	0.003	0.0183	0.0175	0.2954
Body mass index	rs11688816	2	EHBP1	G	0.017	0.003	-0.0159	0.0153	0.3001
Body mass index	rs13021737	2	TMEM18	G	0.060	0.004	-0.0352	0.0201	0.0791
Body mass index	rs1528435	2	UBE2E3	T	0.018	0.003	0.0081	0.0166	0.6258
Body mass index	rs2121279	2	LRP1B	T	0.025	0.004	0.0173	0.0235	0.4610
Body mass index	rs7599312	2	ERBB4	G	0.022	0.003	-0.0358	0.0177	0.0434
Body mass index	rs13078960	3	CADM2	G	0.030	0.004	-0.0259	0.0193	0.1792
Body mass index	rs1516725	3	ETV5	C	0.045	0.005	0.0308	0.0227	0.1761
Body mass index	rs16851483	3	RASA2	T	0.048	0.008	0.0249	0.0328	0.4489
Body mass index	rs2365389	3	FHIT	C	0.020	0.003	-0.0015	0.0158	0.9224
Body mass index	rs3849570	3	GBE1	A	0.019	0.003	0.0319	0.0162	0.0489
Body mass index	rs6804842	3	RARB	G	0.019	0.003	0.0066	0.0158	0.6750
Body mass index	rs10938397	4	GNPDA2	G	0.040	0.003	0.0046	0.0160	0.7740
Body mass index	rs11727676	4	HHIP	T	0.036	0.006	0.0159	0.0419	0.7044
Body mass index	rs13107325	4	SLC39A8	T	0.048	0.007	0.0509	0.0308	0.0983
Body mass index	rs17001654	4	SCARB2	G	0.031	0.005	-0.0018	0.0223	0.9342
Body mass index	rs2112347	5	POC5	T	0.026	0.003	-0.0208	0.0210	0.3241
Body mass index	rs13191362	6	PARK2	A	0.028	0.005	0.0222	0.0237	0.3473
Body mass index	rs2033529	6	TDRG1	G	0.019	0.003	-0.0103	0.0169	0.5434
Body mass index	rs205262	6	C6orf106	G	0.022	0.004	0.0069	0.0171	0.6883
Body mass index	rs2207139	6	TFAP2B	G	0.045	0.004	-0.0001	0.0207	0.9946
Body mass index	rs9400239	6	FOXO3	C	0.019	0.003	0.0348	0.0169	0.0398
Body mass index	rs1167827	7	HIP1	G	0.020	0.003	-0.0042	0.0159	0.7909
Body mass index	rs7792906	7	PMS2L11	C	0.032	0.006	0.0229	0.0344	0.5045
Body mass index	rs17405819	8	HNF4G	T	0.022	0.003	-0.0018	0.0168	0.9162

Body mass index	rs2033732	8	RALYL	C	0.019	0.004	-0.0077	0.0189	0.6833
Body mass index	rs10733682	9	LMX1B	A	0.017	0.003	0.0122	0.0154	0.4287
Body mass index	rs10968576	9	LINGO2	G	0.025	0.003	0.0383	0.0165	0.0205
Body mass index	rs1928295	9	TLR4	T	0.019	0.003	-0.0162	0.0159	0.3077
Body mass index	rs4740619	9	C9orf93	T	0.018	0.003	0.0131	0.0159	0.4082
Body mass index	rs6477694	9	EPB41L4B	C	0.017	0.003	0.0207	0.0167	0.2159
Body mass index	rs11191560	10	NT5C2	C	0.031	0.005	-0.0232	0.0282	0.4101
Body mass index	rs17094222	10	HIF1AN	C	0.025	0.004	-0.0114	0.0193	0.5564
Body mass index	rs7899106	10	GRID1	G	0.040	0.007	0.0256	0.0378	0.4975
Body mass index	rs7903146	10	TCF7L2	C	0.023	0.003	0.0054	0.0173	0.7532
Body mass index	rs11030104	11	BDNF	A	0.041	0.004	-0.0185	0.0186	0.3202
Body mass index	rs12286929	11	CADM1	G	0.022	0.003	-0.0059	0.0154	0.7008
Body mass index	rs2176598	11	HSD17B12	T	0.020	0.004	0.0079	0.0179	0.6599
Body mass index	rs3817334	11	MTCH2	T	0.026	0.003	-0.0616	0.0158	0.00009
Body mass index	rs4256980	11	TRIM66	G	0.021	0.003	0.0228	0.0166	0.1704
Body mass index	rs11057405	12	CLIP1	G	0.031	0.006	0.0121	0.0286	0.6712
Body mass index	rs7138803	12	BCDIN3D	A	0.032	0.003	0.0024	0.0164	0.8825
Body mass index	rs12429545	13	OLFM4	A	0.033	0.005	0.0077	0.0237	0.7451
Body mass index	rs9581854	13	MTIF3	T	0.030	0.005	-0.0120	0.0205	0.5571
Body mass index	rs10132280	14	STXBP6	C	0.023	0.003	0.0053	0.0173	0.7571
Body mass index	rs11847697	14	PRKD1	T	0.049	0.008	-0.0034	0.0400	0.9317
Body mass index	rs12885454	14	PRKD1	C	0.021	0.003	0.0065	0.0164	0.6909
Body mass index	rs7141420	14	NRXN3	T	0.024	0.003	-0.0240	0.0158	0.1290
Body mass index	rs16951275	15	MAP2K5	T	0.031	0.004	0.0145	0.0183	0.4283
Body mass index	rs3736485	15	DMXL2	A	0.018	0.003	0.0148	0.0156	0.3421
Body mass index	rs12446632	16	GPRC5B	G	0.040	0.005	0.0791	0.0227	0.0005
Body mass index	rs1558902	16	FTO	A	0.082	0.003	0.0030	0.0160	0.8507
Body mass index	rs2650492	16	SBK1	A	0.021	0.004	-0.0153	0.0174	0.3773
Body mass index	rs3888190	16	ATP2A1	A	0.031	0.003	-0.0227	0.0162	0.1615
Body mass index	rs758747	16	NLRC3	T	0.023	0.004	0.0196	0.0190	0.3017
Body mass index	rs9925964	16	KAT8	A	0.019	0.003	-0.0403	0.0158	0.0109
Body mass index	rs1000940	17	RABEP1	G	0.019	0.003	0.0463	0.0168	0.0058
Body mass index	rs12940622	17	RPTOR	G	0.018	0.003	-0.0178	0.0154	0.2487
Body mass index	rs1808579	18	C18orf8	C	0.017	0.003	-0.0170	0.0160	0.2876
Body mass index	rs6567160	18	MC4R	C	0.056	0.004	-0.0354	0.0184	0.0544
Body mass index	rs7243357	18	GRP	T	0.022	0.004	0.0109	0.0204	0.5957
Body mass index	rs17724992	19	PGPEP1	A	0.019	0.004	0.0080	0.0177	0.6504
Body mass index	rs2075650	19	TOMM40/APOE	A	0.026	0.005	-1.0415	0.0226	5.1×10^{-464}
Body mass index	rs2287019	19	QPCTL	C	0.036	0.004	-0.0134	0.0209	0.5209
Body mass index	rs29941	19	KCTD15	G	0.018	0.003	0.0011	0.0165	0.9468
Body mass index	rs3810291	19	ZC3H4	A	0.028	0.004	-0.0197	0.0181	0.2768
WHRadjBMI	rs10919388	1	GORAB	C	0.0239	0.0040	0.0126	0.0172	0.4615
WHRadjBMI	rs2645294	1	TBX15-WARS2	T	0.0312	0.0035	-0.0039	0.0161	0.8087
WHRadjBMI	rs2820443	1	LYPLAL1	T	0.0349	0.0037	-0.0403	0.0178	0.0239
WHRadjBMI	rs714515	1	DNM3-PIGC	G	0.0270	0.0034	0.0020	0.0155	0.8963
WHRadjBMI	rs905938	1	DCST2	T	0.0249	0.0040	-0.0003	0.0182	0.9887
WHRadjBMI	rs10195252	2	GRB14-COBLL1	T	0.0270	0.0035	0.0265	0.0162	0.1020
WHRadjBMI	rs1385167	2	MEIS1	G	0.0293	0.0049	-0.0473	0.0235	0.0443

WHRadjBMI	rs1569135	2	CALCRL	A	0.0213	0.0034	0.0189	0.0153	0.2150
WHRadjBMI	rs10804591	3	PLXND1	A	0.0245	0.0042	0.0493	0.0186	0.0081
WHRadjBMI	rs17451107	3	LEKR1	T	0.0256	0.0036	-0.0134	0.0161	0.4055
WHRadjBMI	rs17819328	3	PPARG	G	0.0208	0.0035	-0.0014	0.0164	0.9331
WHRadjBMI	rs2276824	3	PBRM1	C	0.0236	0.0036	0.0066	0.0163	0.6853
WHRadjBMI	rs2371767	3	ADAMTS9	G	0.0363	0.0039	0.0442	0.0173	0.0105
WHRadjBMI	rs303084	4	SPATA5-FGF2	A	0.0232	0.0042	-0.0129	0.0187	0.4923
WHRadjBMI	rs9991328	4	FAM13A	T	0.0185	0.0034	0.0066	0.0158	0.6787
WHRadjBMI	rs6556301	5	FGFR4	T	0.0221	0.0040	0.0103	0.0168	0.5396
WHRadjBMI	rs7705502	5	CPEB4	A	0.0271	0.0036	0.0097	0.0170	0.5688
WHRadjBMI	rs1294410	6	LY86	C	0.0309	0.0035	-0.0068	0.0162	0.6730
WHRadjBMI	rs1358980	6	VEGFA	T	0.0392	0.0036	-0.0270	0.0168	0.1072
WHRadjBMI	rs1936805	6	RSPO3	T	0.0425	0.0034	0.0091	0.0156	0.5586
WHRadjBMI	rs10245353	7	NFE2L3	A	0.0348	0.0043	-0.0092	0.0208	0.6594
WHRadjBMI	rs7801581	7	HOXA11	T	0.0265	0.0042	-0.0049	0.0191	0.7991
WHRadjBMI	rs12679556	8	MSC	G	0.0268	0.0040	-0.0079	0.0184	0.6663
WHRadjBMI	rs10991437	9	ABCA1	A	0.0311	0.0054	-0.0035	0.0244	0.8856
WHRadjBMI	rs11231693	11	MACROD1-VEGFB	A	0.0411	0.0075	0.0107	0.0341	0.7546
WHRadjBMI	rs10842707	12	ITPR2-SSPN	T	0.0324	0.0040	0.0066	0.0187	0.7256
WHRadjBMI	rs1443512	12	HOXC13	A	0.0283	0.0039	0.0383	0.0183	0.0361
WHRadjBMI	rs4765219	12	CCDC92	C	0.0284	0.0036	-0.0081	0.0167	0.6267
WHRadjBMI	rs1440372	15	SMAD6	C	0.0244	0.0038	0.0085	0.0176	0.6275
WHRadjBMI	rs8030605	15	RFX7	A	0.0304	0.0053	0.0181	0.0229	0.4302
WHRadjBMI	rs8042543	15	KLF13	C	0.0263	0.0043	0.0034	0.0202	0.8671
WHRadjBMI	rs4646404	17	PEMT	G	0.0266	0.0039	-0.004	0.0168	0.8095
WHRadjBMI	rs12608504	19	JUND	A	0.0219	0.0036	0.0055	0.0160	0.7309
WHRadjBMI	rs4081724	19	CEBPA	G	0.0347	0.0051	0.0272	0.0224	0.2247
WHRadjBMI	rs224333	20	GDF5	G	0.0202	0.0036	0.0184	0.0168	0.2733
WHRadjBMI	rs6090583	20	EYA2	A	0.0224	0.0034	0.0062	0.0155	0.6877
WHRadjBMI	rs979012	20	BMP2	T	0.0273	0.0036	0.0497	0.0166	0.0028
WHRadjBMI	rs2294239	22	ZNRF3-KREMEN1	A	0.0252	0.0035	-0.0066	0.0155	0.6694
Type 2 diabetes	rs10923931	1	NOTCH2	T	0.077	0.019	0.0041	0.0251	0.8700
Type 2 diabetes	rs2075423	1	PROX1	G	0.068	0.012	-0.0377	0.0167	0.0241
Type 2 diabetes	rs10203174	2	THADA	C	0.131	0.020	0.0671	0.0247	0.0067
Type 2 diabetes	rs13389219	2	GRB14	C	0.068	0.012	0.0271	0.0159	0.0887
Type 2 diabetes	rs243088	2	BCL11A	T	0.068	0.012	-0.0008	0.0156	0.9598
Type 2 diabetes	rs2943640	2	IRS1	C	0.095	0.023	0.0086	0.0160	0.5896
Type 2 diabetes	rs780094	2	GCKR	C	0.058	0.012	0.0027	0.0161	0.8673
Type 2 diabetes	rs11717195	3	ADCY5	T	0.104	0.014	0.0313	0.0185	0.0905
Type 2 diabetes	rs1496653	3	UBE2E2	A	0.086	0.014	-0.0041	0.0193	0.8327
Type 2 diabetes	rs1801282	3	PPARG	C	0.122	0.018	0.0281	0.0237	0.2367
Type 2 diabetes	rs4402960	3	IGF2BP2	T	0.122	0.014	0.0144	0.0171	0.3984
Type 2 diabetes	rs6795735	3	ADAMTS9	C	0.077	0.012	0.0176	0.0162	0.2759
Type 2 diabetes	rs4458523	4	WFS1	G	0.095	0.012	0.0268	0.0159	0.0916
Type 2 diabetes	rs459193	5	ANKRD55	G	0.077	0.014	-0.0007	0.0185	0.9681
Type 2 diabetes	rs7708285	5	ZBED3	G	0.095	0.014	0.0067	0.0176	0.7056
Type 2 diabetes	rs7756992	6	CDKAL1	G	0.157	0.013	-0.0078	0.0171	0.6504
Type 2 diabetes	rs10278336	7	GCK	A	0.068	0.014	-0.0048	0.0162	0.7657

Type 2 diabetes	rs13233731	7	KLF14	G	0.049	0.012	-0.0030	0.0164	0.8536
Type 2 diabetes	rs17168486	7	DGKB	T	0.104	0.016	-0.0254	0.0215	0.2364
Type 2 diabetes	rs849135	7	JAZF1	G	0.104	0.012	-0.0418	0.0155	0.0071
Type 2 diabetes	rs3802177	8	SLC30A8	G	0.131	0.013	0.0334	0.0171	0.0508
Type 2 diabetes	rs516946	8	ANK1	C	0.086	0.014	-0.0122	0.0179	0.4957
Type 2 diabetes	rs7845219	8	TP53INP1	T	0.058	0.012	-0.0485	0.0160	0.0024
Type 2 diabetes	rs10811661	9	CDKN2A/B	T	0.166	0.015	-0.0059	0.0207	0.7759
Type 2 diabetes	rs17791513	9	TLE4	A	0.113	0.023	0.0034	0.0336	0.9189
Type 2 diabetes	rs2796441	9	TLE1	G	0.068	0.012	-0.014	0.0170	0.4106
Type 2 diabetes	rs1111875	10	HHEX/IDE	C	0.104	0.014	0.0010	0.0167	0.9522
Type 2 diabetes	rs11257655	10	CDC123/CAMK1D	T	0.068	0.014	0.0041	0.0198	0.8356
Type 2 diabetes	rs12571751	10	ZMIZ1	A	0.077	0.012	0.0331	0.0160	0.0380
Type 2 diabetes	rs7903146	10	TCF7L2	T	0.329	0.013	-0.0054	0.0173	0.7532
Type 2 diabetes	rs10830963	11	MTNR1B	G	0.095	0.014	0.0146	0.0193	0.4473
Type 2 diabetes	rs1552224	11	ARAP1 (CENTD2)	A	0.104	0.016	-0.005	0.0218	0.8171
Type 2 diabetes	rs163184	11	KCNQ1	G	0.086	0.012	0.0186	0.0159	0.2432
Type 2 diabetes	rs2334499	11	HCCA2	T	0.039	0.010	0.0268	0.0164	0.1015
Type 2 diabetes	rs5215	11	KCNJ11	C	0.068	0.012	0.0291	0.0165	0.0778
Type 2 diabetes	rs10842994	12	KLHDC5	C	0.095	0.016	0.0191	0.0206	0.3540
Type 2 diabetes	rs11063069	12	CCND2	G	0.077	0.014	0.0443	0.0271	0.1021
Type 2 diabetes	rs12427353	12	HNF1A (TCF1)	G	0.077	0.016	0.0032	0.0199	0.8711
Type 2 diabetes	rs2261181	12	HMGA2	T	0.122	0.020	-0.0287	0.0258	0.2662
Type 2 diabetes	rs7955901	12	TSPAN8/LGR5	C	0.068	0.012	-0.0140	0.0159	0.3785
Type 2 diabetes	rs1359790	13	SPRY2	G	0.077	0.012	-0.0004	0.0178	0.9803
Type 2 diabetes	rs11634397	15	ZFAND6	G	0.049	0.012	-0.0071	0.0178	0.6886
Type 2 diabetes	rs12899811	15	PRC1	G	0.077	0.012	0.0213	0.0165	0.1981
Type 2 diabetes	rs7177055	15	HMG20A	A	0.077	0.012	-0.0129	0.0170	0.4458
Type 2 diabetes	rs7202877	16	BCAR1	T	0.113	0.021	-0.0407	0.0270	0.1317
Type 2 diabetes	rs9936385	16	FTO	C	0.122	0.014	0.0057	0.0159	0.7177
Type 2 diabetes	rs4430796	17	HNF1B (TCF2)	G	0.122	0.027	0.0056	0.0169	0.7385
Type 2 diabetes	rs12970134	18	MC4R	A	0.077	0.014	-0.0352	0.0175	0.0444
Type 2 diabetes	rs10401969	19	CILP2	C	0.122	0.020	-0.0163	0.0346	0.6368
Type 2 diabetes	rs8108269	19	GIPR	G	0.068	0.014	-0.0367	0.0176	0.0371
Fasting glucose	rs340874	1	PROX1	C	0.013	0.002	-0.0372	0.0161	0.0213
Fasting glucose	rs560887	2	G6PC2	C	0.071	0.002	0.0232	0.0174	0.1822
Fasting glucose	rs780094	2	GCKR	C	0.027	0.002	0.0027	0.0161	0.8673
Fasting glucose	rs11708067	3	ADCY5	A	0.023	0.003	0.0354	0.0189	0.0613
Fasting glucose	rs11715915	3	AMT	C	0.012	0.002	0.0102	0.0175	0.5586
Fasting glucose	rs11920090	3	SLC2A2	T	0.026	0.003	-0.0381	0.0224	0.0894
Fasting glucose	rs7651090	3	IGF2BP2	G	0.013	0.002	0.0150	0.0166	0.3653
Fasting glucose	rs4869272	5	PCSK1	T	0.018	0.002	0.0072	0.0171	0.6729
Fasting glucose	rs7708285	5	ZBED3	G	0.015	0.003	0.0067	0.0176	0.7056
Fasting glucose	rs17762454	6	RREB1	T	0.014	0.002	0.0034	0.0179	0.8499
Fasting glucose	rs9368222	6	CDKAL1	A	0.014	0.002	-0.0155	0.0178	0.3857
Fasting glucose	rs2191349	7	DGKB/TMEM195	T	0.029	0.002	0.0186	0.0156	0.2339
Fasting glucose	rs4607517	7	GCK	A	0.057	0.003	0.0118	0.0202	0.5583
Fasting glucose	rs6943153	7	GRB10	T	0.015	0.002	0.0032	0.0173	0.8554
Fasting glucose	rs11558471	8	SLC30A8	A	0.029	0.002	0.0323	0.0171	0.0595

Fasting glucose	rs983309	8	PPP1R3B	T	0.026	0.003	0.0237	0.0255	0.3539
Fasting glucose	rs10811661	9	CDKN2B	T	0.024	0.003	-0.0059	0.0207	0.7759
Fasting glucose	rs16913693	9	IKBKAP	T	0.043	0.007	-0.0487	0.0615	0.4280
Fasting glucose	rs3829109	9	LOC728489	G	0.017	0.003	0.0439	0.0199	0.0273
Fasting glucose	rs7867224	9	GLIS3	A	0.013	0.002	0.0137	0.0156	0.3784
Fasting glucose	rs10885122	10	ADRA2A	G	0.027	0.003	0.0059	0.0244	0.8100
Fasting glucose	rs4506565	10	TCF7L2	T	0.021	0.002	-0.008	0.0166	0.6295
Fasting glucose	rs10830963	11	MTNR1B	G	0.078	0.002	0.0146	0.0193	0.4473
Fasting glucose	rs11603334	11	ARAP1	G	0.019	0.003	-0.0008	0.0221	0.9725
Fasting glucose	rs11605924	11	CRY2	A	0.020	0.002	0.0428	0.0156	0.0060
Fasting glucose	rs174550	11	FADS1	T	0.019	0.002	0.0124	0.0163	0.4451
Fasting glucose	rs7944584	11	MADD	A	0.023	0.002	-0.0796	0.0171	3.0×10^{-6}
Fasting glucose	rs10747083	12	P2RX2	A	0.013	0.002	0.0317	0.0207	0.1260
Fasting glucose	rs2657879	12	GLS2	G	0.016	0.003	0.0467	0.0207	0.0242
Fasting glucose	rs11619319	13	PDX1	G	0.019	0.002	-0.0149	0.0191	0.4356
Fasting glucose	rs576674	13	KL	G	0.017	0.003	0.0123	0.0208	0.5542
Fasting glucose	rs3783347	14	WARS	G	0.017	0.003	-0.0125	0.0188	0.5057
Fasting glucose	rs11071657	15	VPS13C/C2CD4A/B	A	0.010	0.002	-0.0104	0.0165	0.5286
Fasting glucose	rs2302593	19	GIPR	C	0.014	0.002	-0.0582	0.0164	0.0004
Fasting glucose	rs6072275	20	TOP1	A	0.016	0.003	0.0184	0.0216	0.3931
Fasting glucose	rs6113722	20	FOXA2	G	0.035	0.005	0.0189	0.0387	0.6256
Fasting insulin	rs4846565	1	LYPLAL1	G	0.013	0.002	-0.0259	0.0166	0.1186
Fasting insulin	rs10195252	2	GRB14	T	0.016	0.003	0.0265	0.0162	0.1020
Fasting insulin	rs1530559	2	YSK4	A	0.015	0.003	-0.0357	0.0191	0.0618
Fasting insulin	rs2943645	2	IRS1	T	0.019	0.002	0.0106	0.0164	0.5199
Fasting insulin	rs780094	2	GCKR	C	0.019	0.002	0.0027	0.0161	0.8673
Fasting insulin	rs17036328	3	PPARG	T	0.021	0.003	0.0280	0.0244	0.2512
Fasting insulin	rs3822072	4	FAM13A1	A	0.012	0.002	0.0096	0.0157	0.5413
Fasting insulin	rs6822892	4	PDGFC	A	0.014	0.002	-0.0086	0.0162	0.5981
Fasting insulin	rs974801	4	TET2	G	0.014	0.002	0.0141	0.0158	0.3716
Fasting insulin	rs459193	5	ANKRD55	G	0.015	0.002	-0.0007	0.0185	0.9681
Fasting insulin	rs4865796	5	ARL15	A	0.015	0.003	0.0123	0.0167	0.4635
Fasting insulin	rs2745353	6	RSPO3	T	0.014	0.002	0.0089	0.0156	0.5681
Fasting insulin	rs6912327	6	C6orf107	T	0.017	0.003	-0.0059	0.0193	0.7618
Fasting insulin	rs1167800	7	HIP1	A	0.016	0.003	-0.009	0.0163	0.5802
Fasting insulin	rs2126259	8	PPP1R3B	T	0.024	0.003	0.0054	0.0273	0.8446
Fasting insulin	rs7903146	10	TCF7L2	C	0.018	0.003	0.0054	0.0173	0.7532
Fasting insulin	rs860598	12	IGF1	A	0.018	0.003	0.0111	0.0207	0.5941
Fasting insulin	rs1421085	16	FTO	C	0.020	0.003	-0.0005	0.0163	0.9763
Fasting insulin	rs731839	19	PEPD	G	0.015	0.003	0.0102	0.0168	0.5428
Systolic BP	rs2404715	1	NA	C	0.400	NA	0.0065	0.0269	0.8095
Systolic BP	rs60199046	1	NA	A	0.240	NA	-0.0083	0.0174	0.6346
Systolic BP	rs6664664	1	NA	G	0.257	NA	-0.0146	0.0154	0.3446
Systolic BP	rs783621	1	NA	A	0.289	NA	-0.0141	0.0160	0.3778
Systolic BP	rs786919	1	NA	A	0.262	NA	-0.0143	0.0156	0.3580
Systolic BP	rs1063281	2	NA	C	0.247	NA	-0.0275	0.0170	0.1044
Systolic BP	rs1250247	2	NA	C	0.310	NA	-0.0089	0.0188	0.6369
Systolic BP	rs13024657	2	NA	T	0.296	NA	0.0132	0.0218	0.5432

Systolic BP	rs13403122	2	NA	C	0.250	NA	0.0011	0.0175	0.9490
Systolic BP	rs13420463	2	NA	A	0.282	NA	-0.0296	0.0187	0.1130
Systolic BP	rs2360970	2	NA	C	0.215	NA	-0.0005	0.0155	0.9723
Systolic BP	rs3731818	2	NA	G	0.240	NA	0.0177	0.0167	0.2872
Systolic BP	rs6434404	2	NA	A	0.311	NA	0.0143	0.0168	0.3936
Systolic BP	rs7590201	2	NA	T	0.216	NA	-0.0064	0.0156	0.6830
Systolic BP	rs113161639	3	NA	G	0.451	NA	0.0141	0.0261	0.5888
Systolic BP	rs12630213	3	NA	C	0.276	NA	0.0233	0.0166	0.1615
Systolic BP	rs12636552	3	NA	A	0.241	NA	-0.007	0.0167	0.6734
Systolic BP	rs2178452	3	NA	G	0.259	NA	-0.0395	0.0166	0.0173
Systolic BP	rs4141663	3	NA	C	0.258	NA	0.0254	0.0160	0.1138
Systolic BP	rs6803322	3	NA	C	0.228	NA	0.0244	0.0167	0.1444
Systolic BP	rs75305034	3	NA	T	0.331	NA	0.0142	0.0166	0.3926
Systolic BP	rs9844972	3	NA	C	0.441	NA	-0.072	0.0321	0.0248
Systolic BP	rs12504699	4	NA	G	0.221	NA	-0.0431	0.0161	0.0074
Systolic BP	rs13104866	4	NA	G	0.278	NA	0.0077	0.0156	0.6203
Systolic BP	rs13112725	4	NA	C	0.297	NA	-0.0137	0.0186	0.4601
Systolic BP	rs1878406	4	NA	T	0.321	NA	-0.0444	0.0228	0.0514
Systolic BP	rs4292285	4	NA	T	0.230	NA	-0.0142	0.0157	0.3641
Systolic BP	rs55940751	4	NA	C	0.216	NA	0.0204	0.0156	0.1900
Systolic BP	rs66887589	4	NA	C	0.214	NA	0.0024	0.0156	0.8762
Systolic BP	rs7665304	4	NA	A	0.220	NA	-0.0061	0.0158	0.6980
Systolic BP	rs17286052	5	NA	A	0.360	NA	0.0431	0.0215	0.0454
Systolic BP	rs303343	5	NA	T	0.229	NA	-0.012	0.0157	0.4426
Systolic BP	rs4475250	5	NA	G	0.243	NA	-0.0334	0.0159	0.0355
Systolic BP	rs6595838	5	NA	A	0.267	NA	0.0042	0.0170	0.8031
Systolic BP	rs2050663	6	NA	C	0.229	NA	0.0239	0.0160	0.1345
Systolic BP	rs35410524	6	NA	T	0.305	NA	-0.0259	0.0195	0.1839
Systolic BP	rs4712656	6	NA	C	0.227	NA	0.0284	0.0156	0.0697
Systolic BP	rs12670854	7	NA	A	0.360	NA	0.0319	0.0247	0.1970
Systolic BP	rs17423264	7	NA	C	0.366	NA	0.0116	0.0293	0.6921
Systolic BP	rs6957161	7	NA	A	0.256	NA	0.0216	0.0178	0.2250
Systolic BP	rs112875651	8	NA	G	0.226	NA	0.0153	0.0169	0.3656
Systolic BP	rs11993898	8	NA	C	0.326	NA	0.0083	0.0200	0.6781
Systolic BP	rs1569209	8	NA	T	0.404	NA	-0.0004	0.0273	0.9869
Systolic BP	rs7008914	8	NA	T	0.238	NA	0.0201	0.0190	0.2913
Systolic BP	rs76735299	8	NA	A	0.429	NA	-0.0257	0.0297	0.3857
Systolic BP	rs9773022	8	NA	C	0.244	NA	-0.0028	0.0171	0.8678
Systolic BP	rs10818775	9	NA	C	0.332	NA	0.0059	0.0242	0.8089
Systolic BP	rs28663144	9	NA	C	0.694	NA	0.0431	0.0419	0.3031
Systolic BP	rs7041664	9	NA	A	0.247	NA	0.0463	0.0190	0.0151
Systolic BP	rs1848797	10	NA	A	0.265	NA	-0.0212	0.0157	0.1768
Systolic BP	rs34872471	10	NA	C	0.250	NA	-0.0090	0.0169	0.5964
Systolic BP	rs4551692	10	NA	A	0.419	NA	-0.0261	0.0256	0.3079
Systolic BP	rs9888067	10	NA	G	0.282	NA	0.0045	0.0201	0.8215
Systolic BP	rs1245113	11	NA	G	0.355	NA	0.0010	0.0156	0.9491
Systolic BP	rs1938598	11	NA	T	0.330	NA	-0.0331	0.0182	0.0691
Systolic BP	rs2289125	11	NA	C	0.274	NA	-0.0184	0.0204	0.3678

Systolic BP	rs2585810	11	NA	A	0.220	NA	0.0083	0.0161	0.6050
Systolic BP	rs360158	11	NA	A	0.301	NA	-0.0234	0.0162	0.1489
Systolic BP	rs61448762	11	NA	G	0.358	NA	-0.0400	0.0267	0.1339
Systolic BP	rs685149	11	NA	G	0.311	NA	-0.0118	0.0164	0.4728
Systolic BP	rs7107356	11	NA	G	0.309	NA	0.0695	0.0156	8.4×10^{-6}
Systolic BP	rs74237369	11	NA	G	0.315	NA	-0.0304	0.0252	0.2276
Systolic BP	rs7927515	11	NA	A	0.235	NA	-0.0018	0.0163	0.9118
Systolic BP	rs7951348	11	NA	T	0.252	NA	-0.0238	0.0160	0.1382
Systolic BP	rs10747570	12	NA	A	0.256	NA	-0.0021	0.0164	0.9000
Systolic BP	rs10784502	12	NA	T	0.235	NA	0.0249	0.0157	0.1128
Systolic BP	rs11168244	12	NA	C	0.318	NA	0.0041	0.0196	0.8326
Systolic BP	rs17210898	12	NA	G	0.556	NA	0.0217	0.0423	0.6083
Systolic BP	rs7977389	12	NA	T	0.381	NA	0.0001	0.0275	0.9964
Systolic BP	rs63418562	13	NA	C	0.312	NA	-0.0064	0.0183	0.7276
Systolic BP	rs9314907	13	NA	T	0.292	NA	-0.0007	0.0199	0.9722
Systolic BP	rs9565436	13	NA	C	0.303	NA	0.0421	0.0229	0.0665
Systolic BP	rs7161323	14	NA	T	0.275	NA	-0.0323	0.0174	0.0627
Systolic BP	rs8904	14	NA	A	0.259	NA	0.0097	0.0162	0.5502
Systolic BP	rs2759308	15	NA	A	0.276	NA	0.0307	0.0156	0.0498
Systolic BP	rs937213	15	NA	T	0.256	NA	-0.0154	0.0163	0.3450
Systolic BP	rs12596053	16	NA	C	0.275	NA	0.0440	0.0160	0.0060
Systolic BP	rs200541	16	NA	G	0.281	NA	-0.0287	0.0194	0.1389
Systolic BP	rs35261357	16	NA	T	0.265	NA	-0.0217	0.0158	0.1685
Systolic BP	rs9934772	16	NA	T	0.373	NA	-0.0075	0.0155	0.6272
Systolic BP	rs9935770	16	NA	C	0.223	NA	-0.0449	0.0160	0.0051
Systolic BP	rs4295	17	NA	C	0.240	NA	-0.0469	0.0162	0.0039
Systolic BP	rs4788913	17	NA	A	0.283	NA	0.0157	0.0166	0.3420
Systolic BP	rs7225219	17	NA	A	0.248	NA	0.0090	0.0174	0.6028
Systolic BP	rs8073626	17	NA	C	0.217	NA	0.0137	0.0156	0.3793
Systolic BP	rs12606620	18	NA	G	0.292	NA	0.0258	0.0169	0.1275
Systolic BP	rs2193635	18	NA	T	0.274	NA	-0.0135	0.0204	0.5095
Systolic BP	rs8105753	19	NA	A	0.249	NA	0.0165	0.0167	0.3218
Systolic BP	rs6031435	20	NA	G	0.214	NA	0.0218	0.0159	0.1702
Systolic BP	rs6090040	20	NA	A	0.265	NA	-0.0102	0.0187	0.5841
Systolic BP	rs6129880	20	NA	T	0.264	NA	-0.0308	0.0204	0.1323
Systolic BP	rs13050325	21	NA	G	0.258	NA	-0.0296	0.0181	0.1011
Systolic BP	rs34887403	22	NA	A	0.304	NA	-0.0292	0.0228	0.2001
Diastolic BP	rs11102916	1	NA	A	0.575	NA	0.0938	0.0601	0.1187
Diastolic BP	rs11809042	1	NA	G	0.249	NA	0.0280	0.0168	0.0956
Diastolic BP	rs12405515	1	NA	G	0.170	NA	0.0016	0.0227	0.9439
Diastolic BP	rs17046596	1	NA	C	0.151	NA	-0.0345	0.0179	0.0544
Diastolic BP	rs4653889	1	NA	A	0.152	NA	0.0140	0.0160	0.3790
Diastolic BP	rs6428947	1	NA	C	0.205	NA	-0.0385	0.0216	0.0743
Diastolic BP	rs6664664	1	NA	G	0.147	NA	-0.0146	0.0154	0.3446
Diastolic BP	rs10198275	2	NA	A	0.135	NA	-0.0056	0.0158	0.7238
Diastolic BP	rs1063281	2	NA	C	0.170	NA	-0.0275	0.0170	0.1044
Diastolic BP	rs13403122	2	NA	C	0.195	NA	0.0011	0.0175	0.9490
Diastolic BP	rs1867863	2	NA	G	0.143	NA	-0.0062	0.0170	0.7146

Diastolic BP	rs1876487	2	NA	C	0.162	NA	-0.0229	0.0187	0.2202
Diastolic BP	rs3923097	2	NA	T	0.273	NA	-0.008	0.0324	0.8051
Diastolic BP	rs58117425	2	NA	A	0.192	NA	0.0109	0.0186	0.5583
Diastolic BP	rs7590201	2	NA	T	0.138	NA	-0.0064	0.0156	0.6830
Diastolic BP	rs1053711	3	NA	G	0.153	NA	-0.0263	0.0168	0.1179
Diastolic BP	rs113161639	3	NA	G	0.288	NA	0.0141	0.0261	0.5888
Diastolic BP	rs12630213	3	NA	C	0.153	NA	0.0233	0.0166	0.1615
Diastolic BP	rs2236973	3	NA	C	0.182	NA	0.0092	0.0236	0.6964
Diastolic BP	rs3749237	3	NA	A	0.165	NA	-0.0012	0.0166	0.9413
Diastolic BP	rs4686683	3	NA	G	0.172	NA	0.0149	0.0168	0.3758
Diastolic BP	rs6795735	3	NA	C	0.159	NA	0.0176	0.0162	0.2759
Diastolic BP	rs75305034	3	NA	T	0.256	NA	0.0142	0.0166	0.3926
Diastolic BP	rs76398786	3	NA	T	0.382	NA	0.0591	0.0765	0.4401
Diastolic BP	rs9845655	3	NA	T	0.161	NA	-0.0461	0.0177	0.0092
Diastolic BP	rs9864898	3	NA	T	0.190	NA	-0.0169	0.0214	0.4291
Diastolic BP	rs9882772	3	NA	C	0.133	NA	0.0043	0.0157	0.7850
Diastolic BP	rs13112725	4	NA	C	0.150	NA	-0.0137	0.0186	0.4601
Diastolic BP	rs4292285	4	NA	T	0.154	NA	-0.0142	0.0157	0.3641
Diastolic BP	rs66887589	4	NA	C	0.206	NA	0.0024	0.0156	0.8762
Diastolic BP	rs111304266	5	NA	G	0.370	NA	0.0531	0.0542	0.3273
Diastolic BP	rs168643	5	NA	T	0.139	NA	-0.0113	0.0165	0.4922
Diastolic BP	rs17082391	5	NA	C	0.375	NA	-0.1065	0.0473	0.0243
Diastolic BP	rs17286052	5	NA	A	0.188	NA	0.0431	0.0215	0.0454
Diastolic BP	rs258494	5	NA	C	0.216	NA	0.0245	0.0170	0.1494
Diastolic BP	rs4475250	5	NA	G	0.136	NA	-0.0334	0.0159	0.0355
Diastolic BP	rs7734334	5	NA	A	0.154	NA	-0.0015	0.0166	0.9285
Diastolic BP	rs13205180	6	NA	T	0.143	NA	-0.0164	0.0156	0.2917
Diastolic BP	rs13210963	6	NA	C	0.239	NA	-0.0027	0.0183	0.8814
Diastolic BP	rs1544935	6	NA	T	0.176	NA	-0.0179	0.0193	0.3543
Diastolic BP	rs1630266	6	NA	A	0.252	NA	-0.0262	0.0280	0.3494
Diastolic BP	rs169287	6	NA	C	0.215	NA	-0.0034	0.0224	0.8808
Diastolic BP	rs1761870	6	NA	G	0.175	NA	0.0008	0.0205	0.9707
Diastolic BP	rs2050663	6	NA	C	0.174	NA	0.0239	0.0160	0.1345
Diastolic BP	rs210156	6	NA	G	0.148	NA	-0.0214	0.0168	0.2015
Diastolic BP	rs36061333	6	NA	C	0.160	NA	-0.0173	0.0187	0.3554
Diastolic BP	rs4709746	6	NA	C	0.190	NA	-0.0126	0.0243	0.6033
Diastolic BP	rs903432	6	NA	A	0.313	NA	0.0300	0.0322	0.3516
Diastolic BP	rs1015538	7	NA	A	0.137	NA	-0.0544	0.0165	0.0010
Diastolic BP	rs111630016	7	NA	C	0.324	NA	-0.0632	0.0472	0.1809
Diastolic BP	rs11486794	7	NA	C	0.219	NA	-0.0005	0.0231	0.9814
Diastolic BP	rs11556924	7	NA	C	0.181	NA	0.0129	0.0176	0.4640
Diastolic BP	rs34594435	7	NA	C	0.183	NA	-0.0196	0.0203	0.3346
Diastolic BP	rs6957161	7	NA	A	0.157	NA	0.0216	0.0178	0.2250
Diastolic BP	rs76627715	7	NA	T	0.216	NA	0.0123	0.0240	0.6075
Diastolic BP	rs10103353	8	NA	C	0.147	NA	0.0035	0.0162	0.8272
Diastolic BP	rs2280861	8	NA	G	0.173	NA	0.0228	0.0181	0.2064
Diastolic BP	rs9773022	8	NA	C	0.147	NA	-0.0028	0.0171	0.8678
Diastolic BP	rs507666	9	NA	G	0.169	NA	0.0022	0.0194	0.9095

Diastolic BP	rs10751962	10	NA	T	0.219	NA	-0.0300	0.0265	0.2579
Diastolic BP	rs17617337	10	NA	C	0.172	NA	-0.0166	0.0191	0.3842
Diastolic BP	rs1848797	10	NA	A	0.191	NA	-0.0212	0.0157	0.1768
Diastolic BP	rs2246438	10	NA	G	0.160	NA	0.0111	0.0172	0.5182
Diastolic BP	rs4551692	10	NA	A	0.255	NA	-0.0261	0.0256	0.3079
Diastolic BP	rs6479908	10	NA	G	0.142	NA	0.0036	0.0155	0.8160
Diastolic BP	rs11021221	11	NA	T	0.213	NA	-0.0045	0.0222	0.8391
Diastolic BP	rs11030119	11	NA	G	0.180	NA	-0.0225	0.0176	0.2014
Diastolic BP	rs12787709	11	NA	A	0.138	NA	0.0211	0.0171	0.2167
Diastolic BP	rs360158	11	NA	A	0.165	NA	-0.0234	0.0162	0.1489
Diastolic BP	rs628224	11	NA	A	0.198	NA	-0.013	0.0227	0.5675
Diastolic BP	rs7107356	11	NA	G	0.149	NA	0.0695	0.0156	8.4×10^{-6}
Diastolic BP	rs7116797	11	NA	A	0.214	NA	0.0463	0.0260	0.0752
Diastolic BP	rs72930293	11	NA	C	0.234	NA	0.0440	0.0253	0.0817
Diastolic BP	rs7928655	11	NA	G	0.143	NA	0.0227	0.0170	0.1808
Diastolic BP	rs10747570	12	NA	A	0.184	NA	-0.0021	0.0164	0.9000
Diastolic BP	rs11168244	12	NA	C	0.165	NA	0.0041	0.0196	0.8326
Diastolic BP	rs1152958	12	NA	G	0.138	NA	-0.0121	0.0167	0.4680
Diastolic BP	rs17210898	12	NA	G	0.351	NA	0.0217	0.0423	0.6083
Diastolic BP	rs7980687	12	NA	G	0.196	NA	0.0357	0.0196	0.0682
Diastolic BP	rs3934941	13	NA	A	0.163	NA	-0.0062	0.0181	0.7325
Diastolic BP	rs63418562	13	NA	C	0.240	NA	-0.0064	0.0183	0.7276
Diastolic BP	rs36226649	14	NA	C	0.297	NA	0.0292	0.0359	0.4158
Diastolic BP	rs7161323	14	NA	T	0.147	NA	-0.0323	0.0174	0.0627
Diastolic BP	rs11631778	15	NA	A	0.141	NA	-0.001	0.0162	0.9506
Diastolic BP	rs12906962	15	NA	C	0.160	NA	0.0065	0.0173	0.7080
Diastolic BP	rs2034618	15	NA	C	0.188	NA	0.0112	0.0185	0.5452
Diastolic BP	rs2759308	15	NA	A	0.136	NA	0.0307	0.0156	0.0498
Diastolic BP	rs35654783	15	NA	T	0.166	NA	-0.0245	0.0175	0.1606
Diastolic BP	rs4923910	15	NA	C	0.178	NA	-0.0096	0.0165	0.5601
Diastolic BP	rs4984497	15	NA	T	0.148	NA	-0.0163	0.0169	0.3335
Diastolic BP	rs12596053	16	NA	C	0.162	NA	0.0440	0.0160	0.0060
Diastolic BP	rs12928482	16	NA	G	0.147	NA	0.0146	0.0176	0.4047
Diastolic BP	rs460105	16	NA	T	0.178	NA	-0.0167	0.0235	0.4766
Diastolic BP	rs72799341	16	NA	A	0.161	NA	-0.0549	0.0184	0.0028
Diastolic BP	rs35565381	17	NA	C	0.140	NA	0.0163	0.0159	0.3055
Diastolic BP	rs4295	17	NA	C	0.140	NA	-0.0469	0.0162	0.0039
Diastolic BP	rs67833703	17	NA	T	0.147	NA	-0.0266	0.0170	0.1172
Diastolic BP	rs34331990	19	NA	G	0.154	NA	0.0192	0.0163	0.2385
Diastolic BP	rs4808569	19	NA	C	0.162	NA	-0.0104	0.0202	0.6079
Diastolic BP	rs8105753	19	NA	A	0.137	NA	0.0165	0.0167	0.3218
Diastolic BP	rs6019378	20	NA	C	0.156	NA	-0.0161	0.0155	0.2977
Diastolic BP	rs6060114	20	NA	T	0.237	NA	0.0043	0.0207	0.8362
Diastolic BP	rs6129880	20	NA	T	0.174	NA	-0.0308	0.0204	0.1323
Diastolic BP	rs112204826	21	NA	T	0.395	NA	0.0641	0.0500	0.2000
Diastolic BP	rs8139817	22	NA	C	0.143	NA	0.0301	0.0173	0.0819
Total cholesterol	rs1077514	1	ASAP3	T	0.030	NA	0.0029	0.0239	0.9023
Total cholesterol	rs12027135	1	LDL-CRAP1	T	0.027	NA	0.0096	0.0157	0.5393

Total cholesterol	rs2131925	1	ANGPTL3	T	0.075	NA	-0.0334	0.0164	0.0422
Total cholesterol	rs2479409	1	PCSK9	G	0.054	NA	0.0225	0.0184	0.2214
Total cholesterol	rs2642442	1	MOSC1	T	0.035	NA	0.0148	0.0184	0.4229
Total cholesterol	rs514230	1	IRF2BP2	T	0.039	NA	0.0233	0.0157	0.1379
Total cholesterol	rs629301	1	SORT1	T	0.134	NA	0.0030	0.0186	0.8711
Total cholesterol	rs7515577	1	EVI5	A	0.037	NA	-0.0317	0.0198	0.1091
Total cholesterol	rs10490626	2	INSIG2	G	0.042	NA	0.0451	0.0286	0.1152
Total cholesterol	rs11563251	2	UGT1A1	T	0.037	NA	0.0315	0.0265	0.2342
Total cholesterol	rs11694172	2	FAM117B	G	0.028	NA	0.0049	0.0193	0.7986
Total cholesterol	rs1260326	2	GCKR	T	0.051	NA	-0.0008	0.0161	0.9608
Total cholesterol	rs1367117	2	APOB	A	0.100	NA	0.0057	0.0172	0.7400
Total cholesterol	rs2030746	2	LOC84931	T	0.020	NA	-0.0047	0.0165	0.7756
Total cholesterol	rs2287623	2	ABCB11	G	0.027	NA	0.0210	0.0158	0.1840
Total cholesterol	rs4299376	2	ABCG5/8	G	0.079	NA	-0.018	0.0172	0.2952
Total cholesterol	rs7570971	2	RAB3GAP1	A	0.030	NA	0.0285	0.0176	0.1048
Total cholesterol	rs13315871	3	PXK	G	0.036	NA	0.0030	0.0264	0.9103
Total cholesterol	rs2290159	3	RAF1	G	0.037	NA	-0.0445	0.0187	0.0174
Total cholesterol	rs7640978	3	CMTM6	C	0.038	NA	0.0136	0.0282	0.6293
Total cholesterol	rs6831256	4	LRPAP1	G	0.025	NA	0.0124	0.0157	0.4296
Total cholesterol	rs12916	5	HMGCR	C	0.068	NA	0.0047	0.0164	0.7734
Total cholesterol	rs4530754	5	CSNK1G3	A	0.023	NA	0.0216	0.0155	0.1621
Total cholesterol	rs6882076	5	TIMD4	C	0.051	NA	0.0084	0.0169	0.6190
Total cholesterol	rs114342608	6	HLA	C	0.048	NA	0.1008	0.0226	7.9×10^{-6}
Total cholesterol	rs1564348	6	LPA	C	0.049	NA	0.0074	0.0219	0.7366
Total cholesterol	rs1800562	6	HFE	G	0.056	NA	0.0171	0.0328	0.6022
Total cholesterol	rs2758886	6	KCNK17	A	0.023	NA	-0.0030	0.0166	0.8571
Total cholesterol	rs2814982	6	C6orf106	C	0.044	NA	-0.0008	0.0261	0.9744
Total cholesterol	rs3757354	6	MYLIP	C	0.035	NA	-0.0127	0.0197	0.5209
Total cholesterol	rs9376090	6	HBS1L	T	0.025	NA	-0.0006	0.0180	0.9717
Total cholesterol	rs9488822	6	FRK	T	0.034	NA	0.0198	0.0167	0.2364
Total cholesterol	rs12670798	7	DNAH11	C	0.036	NA	0.0086	0.0190	0.6529
Total cholesterol	rs1997243	7	GPR146	G	0.033	NA	0.0249	0.0210	0.2364
Total cholesterol	rs2072183	7	NPC1L1	C	0.036	NA	-0.0306	0.0223	0.1692
Total cholesterol	rs4722551	7	MIR148A	C	0.029	NA	-0.0221	0.0216	0.3072
Total cholesterol	rs10102164	8	SOX17	A	0.030	NA	0.0135	0.0200	0.4979
Total cholesterol	rs11136341	8	PLEC1	G	0.038	NA	0.0189	0.0180	0.2932
Total cholesterol	rs1495741	8	NAT2	G	0.032	NA	-0.0041	0.0186	0.8269
Total cholesterol	rs2081687	8	CYP7A1	T	0.038	NA	0.0007	0.0163	0.9646
Total cholesterol	rs2954029	8	TRIB1	A	0.062	NA	0.0137	0.0160	0.3908
Total cholesterol	rs4841132	8	PPP1R3B	G	0.078	NA	-0.014	0.0279	0.6147
Total cholesterol	rs1883025	9	ABCA1	C	0.067	NA	-0.0705	0.0182	0.0001
Total cholesterol	rs3780181	9	VLDL-CR	A	0.044	NA	0.0707	0.0306	0.0209
Total cholesterol	rs581080	9	TTC39B	C	0.038	NA	0.0163	0.0208	0.4346
Total cholesterol	rs635634	9	ABO	T	0.069	NA	-0.0055	0.0195	0.7793
Total cholesterol	rs10904908	10	VIM-CUBN	G	0.025	NA	0.0024	0.0159	0.8826
Total cholesterol	rs2255141	10	GPAM	A	0.031	NA	0.0129	0.0168	0.4451
Total cholesterol	rs970548	10	MARCH8-ALOX5	C	0.025	NA	0.0050	0.0178	0.7767
Total cholesterol	rs10128711	11	SPTY2D1	C	0.031	NA	-0.0003	0.0182	0.9877

Total cholesterol	rs11220462	11	ST3GAL4	A	0.047	NA	0.0183	0.0225	0.4154
Total cholesterol	rs11603023	11	PHLDB1	T	0.022	NA	0.0064	0.0161	0.6917
Total cholesterol	rs174546	11	FADS1-2-3	C	0.048	NA	0.0104	0.0168	0.5374
Total cholesterol	rs6589939	11	UBASH3B	G	0.0311	NA	0.0302	0.0161	0.0605
Total cholesterol	rs964184	11	APOA1	G	0.121	NA	0.0207	0.0226	0.3617
Total cholesterol	rs11065987	12	BRAP	A	0.031	NA	0.0123	0.0159	0.4368
Total cholesterol	rs1169288	12	HNF1A	C	0.032	NA	-0.0167	0.0169	0.3213
Total cholesterol	rs4883201	12	PHC1-A2ML1	A	0.035	NA	0.0249	0.0263	0.3451
Total cholesterol	rs1532085	15	LIPC	A	0.054	NA	-0.0390	0.0160	0.0148
Total cholesterol	rs2000999	16	HPR	A	0.062	NA	-0.0082	0.0195	0.6764
Total cholesterol	rs3764261	16	CETP	A	0.050	NA	0.0032	0.0165	0.8462
Total cholesterol	rs314253	17	DLG4	T	0.023	NA	-0.0244	0.0166	0.1417
Total cholesterol	rs7206971	17	OSBPL7	A	0.030	NA	-0.0535	0.0167	0.0014
Total cholesterol	rs7241918	18	LIPG	T	0.058	NA	-0.0149	0.0211	0.4798
Total cholesterol	rs10401969	19	CILP2	T	0.137	NA	0.0163	0.0346	0.6368
Total cholesterol	rs492602	19	FLJ36070	G	0.031	NA	0.0249	0.0162	0.1230
Total cholesterol	rs6511720	19	LDL-CR	G	0.185	NA	0.0095	0.0260	0.7143
Total cholesterol	rs6857	19	APOE	T	0.164	NA	1.1610	0.0226	2.5×10^{-575}
Total cholesterol	rs1800961	20	HNF4A	C	0.106	NA	-0.0596	0.0467	0.2016
Total cholesterol	rs2277862	20	ERGIC3	C	0.035	NA	-0.018	0.0218	0.4102
Total cholesterol	rs2902940	20	MAFB	A	0.024	NA	-0.0112	0.0173	0.5176
Total cholesterol	rs6029526	20	TOP1	A	0.040	NA	0.0084	0.0156	0.5902
Total cholesterol	rs138777	22	TOM1	A	0.021	NA	0.0096	0.0163	0.5554
Total cholesterol	rs4253772	22	PPARA	T	0.032	NA	0.0090	0.0255	0.7240
HDL cholesterol	rs12145743	1	HDGF-PMVK	G	0.020	NA	0.0253	0.0167	0.1290
HDL cholesterol	rs12748152	1	PIGV-NR0B2	C	0.051	NA	-0.0344	0.0295	0.2429
HDL cholesterol	rs1689800	1	ZNF648	A	0.034	NA	-0.0028	0.0160	0.8607
HDL cholesterol	rs4650994	1	ANGPTL1	G	0.021	NA	-0.0040	0.0155	0.7969
HDL cholesterol	rs4660293	1	PABPC4	A	0.035	NA	-0.0181	0.0188	0.3358
HDL cholesterol	rs4846914	1	GALNT2	A	0.048	NA	0.0077	0.0166	0.6404
HDL cholesterol	rs1047891	2	CPS1	C	0.027	NA	0.0154	0.0195	0.4316
HDL cholesterol	rs12328675	2	COBLL1	C	0.045	NA	0.0020	0.0241	0.9350
HDL cholesterol	rs2972146	2	IRS1	G	0.032	NA	-0.0131	0.0159	0.4116
HDL cholesterol	rs13326165	3	STAB1	A	0.029	NA	0.0312	0.0195	0.1088
HDL cholesterol	rs17404153	3	ACAD11	G	0.028	NA	0.0140	0.0245	0.5682
HDL cholesterol	rs2013208	3	RBM5	T	0.025	NA	-0.0174	0.016	0.2780
HDL cholesterol	rs2290547	3	SETD2	G	0.030	NA	0.0015	0.0209	0.9435
HDL cholesterol	rs2606736	3	ATG7	C	0.025	NA	0.0011	0.0165	0.9457
HDL cholesterol	rs6805251	3	GSK3B	T	0.020	NA	-0.0143	0.0157	0.3626
HDL cholesterol	rs10019888	4	C4orf52	A	0.027	NA	0.0022	0.0208	0.9173
HDL cholesterol	rs13107325	4	SLC39A8	C	0.071	NA	-0.0509	0.0308	0.0983
HDL cholesterol	rs2602836	4	ADH5	A	0.019	NA	-0.0242	0.0156	0.1208
HDL cholesterol	rs3822072	4	FAM13A	G	0.025	NA	-0.0096	0.0157	0.5413
HDL cholesterol	rs6450176	5	ARL15	G	0.025	NA	0.0201	0.0176	0.2553
HDL cholesterol	rs1936800	6	RSPO3	C	0.020	NA	-0.0061	0.0157	0.6995
HDL cholesterol	rs605066	6	CITED2	T	0.028	NA	0.0022	0.0158	0.8904
HDL cholesterol	rs998584	6	VEGFA	C	0.026	NA	0.0292	0.0181	0.1061
HDL cholesterol	rs17145738	7	MLXIPL	T	0.041	NA	0.0255	0.0243	0.2943

HDL cholesterol	rs17173637	7	TMEM176A	T	0.036	NA	-0.0143	0.0283	0.6142
HDL cholesterol	rs4142995	7	SNX13	G	0.026	NA	0.0145	0.0161	0.3682
HDL cholesterol	rs4731702	7	KLF14	T	0.029	NA	0.0045	0.0165	0.7841
HDL cholesterol	rs4917014	7	IKZF1	G	0.022	NA	0.0370	0.0168	0.0275
HDL cholesterol	rs702485	7	DAGLB	G	0.024	NA	-0.0486	0.0156	0.0019
HDL cholesterol	rs12678919	8	LPL	G	0.155	NA	0.0105	0.0251	0.6759
HDL cholesterol	rs2293889	8	TRPS1	G	0.031	NA	0.0305	0.0164	0.0622
HDL cholesterol	rs2954029	8	TRIB1	T	0.040	NA	-0.0137	0.0160	0.3908
HDL cholesterol	rs4841132	8	PPP1R3B	G	0.0816	NA	-0.014	0.0279	0.6147
HDL cholesterol	rs1883025	9	ABCA1	C	0.070	NA	-0.0705	0.0182	0.0001
HDL cholesterol	rs581080	9	TTC39B	C	0.042	NA	0.0163	0.0208	0.4346
HDL cholesterol	rs970548	10	MARCH8-ALOX5	C	0.026	NA	0.0050	0.0178	0.7767
HDL cholesterol	rs11246602	11	OR4C46	C	0.034	NA	0.0333	0.0256	0.1933
HDL cholesterol	rs12801636	11	KAT5	A	0.024	NA	-0.0118	0.0185	0.5242
HDL cholesterol	rs174546	11	FADS1-2-3	C	0.039	NA	0.0104	0.0168	0.5374
HDL cholesterol	rs2923084	11	AMPD3	A	0.026	NA	0.0321	0.0200	0.1091
HDL cholesterol	rs3136441	11	LRP4	C	0.054	NA	0.0132	0.0232	0.5708
HDL cholesterol	rs499974	11	MOGAT2-DGAT2	C	0.026	NA	-0.0108	0.0212	0.6101
HDL cholesterol	rs6589939	11	UBASH3B	G	0.0224	NA	0.0302	0.0161	0.0605
HDL cholesterol	rs964184	11	APOA1	C	0.106	NA	-0.0207	0.0226	0.3617
HDL cholesterol	rs11613352	12	LRP1	T	0.028	NA	0.0106	0.0185	0.5644
HDL cholesterol	rs4759375	12	SBNO1	T	0.056	NA	-0.0361	0.0267	0.1756
HDL cholesterol	rs4765127	12	ZNF664	T	0.032	NA	0.0102	0.0161	0.5278
HDL cholesterol	rs7134375	12	PDE3A	A	0.021	NA	-0.0016	0.0159	0.9206
HDL cholesterol	rs7134594	12	MVK	T	0.035	NA	0.0210	0.0158	0.1853
HDL cholesterol	rs838880	12	SCARB1	C	0.048	NA	0.0066	0.0177	0.7092
HDL cholesterol	rs4983559	14	ZBTB42-AKT1	G	0.020	NA	-0.0256	0.0173	0.1394
HDL cholesterol	rs1532085	15	LIPC	A	0.107	NA	-0.0390	0.0160	0.0148
HDL cholesterol	rs2652834	15	LACTB	G	0.028	NA	0.0449	0.0192	0.0197
HDL cholesterol	rs1121980	16	FTO	G	0.020	NA	-0.0005	0.0157	0.9724
HDL cholesterol	rs16942887	16	LCAT	A	0.083	NA	0.0046	0.0237	0.8452
HDL cholesterol	rs2925979	16	CMIP	C	0.035	NA	-0.0063	0.0175	0.7195
HDL cholesterol	rs3764261	16	CETP	A	0.241	NA	0.0032	0.0165	0.8462
HDL cholesterol	rs11869286	17	STARD3	C	0.032	NA	0.0144	0.0165	0.3828
HDL cholesterol	rs4129767	17	PGS1	A	0.024	NA	0.0350	0.0156	0.0245
HDL cholesterol	rs4148008	17	ABCA8	C	0.028	NA	0.0177	0.0164	0.2795
HDL cholesterol	rs12967135	18	MC4R	G	0.026	NA	0.0348	0.0182	0.0562
HDL cholesterol	rs7241918	18	LIPG	T	0.090	NA	-0.0149	0.0211	0.4798
HDL cholesterol	rs17695224	19	HAS1	G	0.029	NA	0.0019	0.0174	0.9126
HDL cholesterol	rs386000	19	LILRA3	C	0.048	NA	0.0207	0.0213	0.3308
HDL cholesterol	rs6857	19	APOE	C	0.067	NA	-1.1610	0.0226	2.5×10^{-575}
HDL cholesterol	rs7255436	19	ANGPTL4	A	0.032	NA	-0.0282	0.0162	0.0824
HDL cholesterol	rs731839	19	PEPD	A	0.022	NA	-0.0102	0.0168	0.5428
HDL cholesterol	rs737337	19	ANGPTL8	T	0.056	NA	-0.0416	0.0311	0.1821
HDL cholesterol	rs1800961	20	HNF4A	C	0.127	NA	-0.0596	0.0467	0.2016
HDL cholesterol	rs6065906	20	PLTP	T	0.059	NA	0.0153	0.0192	0.4275
HDL cholesterol	rs181360	22	UBE2L3	T	0.038	NA	0.0699	0.0203	0.0006
LDL cholesterol	rs12027135	1	LDL-CRAP1	T	0.030	NA	0.0096	0.0157	0.5393

LDL cholesterol	rs12748152	1	PIGV-NR0B2	T	0.050	NA	0.0344	0.0295	0.2429
LDL cholesterol	rs2131925	1	ANGPTL3	T	0.049	NA	-0.0334	0.0164	0.0422
LDL cholesterol	rs2479409	1	PCSK9	G	0.064	NA	0.0225	0.0184	0.2214
LDL cholesterol	rs2642442	1	MOSC1	T	0.036	NA	0.0148	0.0184	0.4229
LDL cholesterol	rs267733	1	ANXA9-CERS2	A	0.033	NA	0.0409	0.0217	0.0593
LDL cholesterol	rs514230	1	IRF2BP2	T	0.036	NA	0.0233	0.0157	0.1379
LDL cholesterol	rs629301	1	SORT1	T	0.167	NA	0.0030	0.0186	0.8711
LDL cholesterol	rs10490626	2	INSIG2	G	0.051	NA	0.0451	0.0286	0.1152
LDL cholesterol	rs11563251	2	UGT1A1	T	0.034	NA	0.0315	0.0265	0.2342
LDL cholesterol	rs1250229	2	FN1	C	0.024	NA	0.0183	0.0188	0.3303
LDL cholesterol	rs1367117	2	APOB	A	0.119	NA	0.0057	0.0172	0.7400
LDL cholesterol	rs2030746	2	LOC84931	T	0.021	NA	-0.0047	0.0165	0.7756
LDL cholesterol	rs2710642	2	EHBP1	A	0.024	NA	0.0046	0.0162	0.7752
LDL cholesterol	rs4299376	2	ABCG5/8	G	0.081	NA	-0.018	0.0172	0.2952
LDL cholesterol	rs17404153	3	ACAD11	G	0.034	NA	0.0140	0.0245	0.5682
LDL cholesterol	rs7640978	3	CMTM6	C	0.039	NA	0.0136	0.0282	0.6293
LDL cholesterol	rs6831256	4	LRPAP1	G	0.022	NA	0.0124	0.0157	0.4296
LDL cholesterol	rs12916	5	HMGCR	C	0.073	NA	0.0047	0.0164	0.7734
LDL cholesterol	rs4530754	5	CSNK1G3	A	0.028	NA	0.0216	0.0155	0.1621
LDL cholesterol	rs6882076	5	TIMD4	C	0.046	NA	0.0084	0.0169	0.6190
LDL cholesterol	rs1564348	6	LPA	C	0.048	NA	0.0074	0.0219	0.7366
LDL cholesterol	rs1800562	6	HFE	G	0.062	NA	0.0171	0.0328	0.6022
LDL cholesterol	rs3757354	6	MYLIP	C	0.038	NA	-0.0127	0.0197	0.5209
LDL cholesterol	rs9488822	6	FRK	T	0.031	NA	0.0198	0.0167	0.2364
LDL cholesterol	rs12670798	7	DNAH11	C	0.034	NA	0.0086	0.0190	0.6529
LDL cholesterol	rs2072183	7	NPC1L1	C	0.039	NA	-0.0306	0.0223	0.1692
LDL cholesterol	rs4722551	7	MIR148A	C	0.039	NA	-0.0221	0.0216	0.3072
LDL cholesterol	rs10102164	8	SOX17	A	0.032	NA	0.0135	0.0200	0.4979
LDL cholesterol	rs11136341	8	PLEC1	G	0.045	NA	0.0189	0.0180	0.2932
LDL cholesterol	rs2081687	8	CYP7A1	T	0.031	NA	0.0007	0.0163	0.9646
LDL cholesterol	rs2954029	8	TRIB1	A	0.056	NA	0.0137	0.0160	0.3908
LDL cholesterol	rs4841132	8	PPP1R3B	G	0.067	NA	-0.014	0.0279	0.6147
LDL cholesterol	rs3780181	9	VLDL-CR	A	0.044	NA	0.0707	0.0306	0.0209
LDL cholesterol	rs635634	9	ABO	T	0.077	NA	-0.0055	0.0195	0.7793
LDL cholesterol	rs2255141	10	GPAM	A	0.030	NA	0.0129	0.0168	0.4451
LDL cholesterol	rs11220462	11	ST3GAL4	A	0.059	NA	0.0183	0.0225	0.4154
LDL cholesterol	rs174546	11	FADS1-2-3	C	0.051	NA	0.0104	0.0168	0.5374
LDL cholesterol	rs964184	11	APOA1	G	0.086	NA	0.0207	0.0226	0.3617
LDL cholesterol	rs11065987	12	BRAP	A	0.027	NA	0.0123	0.0159	0.4368
LDL cholesterol	rs1169288	12	HNF1A	C	0.038	NA	-0.0167	0.0169	0.3213
LDL cholesterol	rs4942486	13	BRCA2	T	0.024	NA	0.0131	0.0159	0.4080
LDL cholesterol	rs8017377	14	NYNRIN	A	0.030	NA	-0.0092	0.0160	0.5678
LDL cholesterol	rs2000999	16	HPR	A	0.065	NA	-0.0082	0.0195	0.6764
LDL cholesterol	rs3764261	16	CETP	C	0.053	NA	-0.0032	0.0165	0.8462
LDL cholesterol	rs1801689	17	APOH-PRXCA	C	0.103	NA	-0.1437	0.0759	0.0583
LDL cholesterol	rs314253	17	DLG4	T	0.024	NA	-0.0244	0.0166	0.1417
LDL cholesterol	rs7206971	17	OSBPL7	A	0.029	NA	-0.0535	0.0167	0.0014
LDL cholesterol	rs10401969	19	CILP2	T	0.118	NA	0.0163	0.0346	0.6368

LDL cholesterol	rs6511720	19	LDL-CR	G	0.221	NA	0.0095	0.026	0.7143
LDL cholesterol	rs6857	19	APOE	T	0.192	NA	1.1610	0.0226	2.5×10^{-575}
LDL cholesterol	rs2328223	20	SNX5	C	0.030	NA	0.0428	0.0198	0.0310
LDL cholesterol	rs2902940	20	MAFB	A	0.027	NA	-0.0112	0.0173	0.5176
LDL cholesterol	rs364585	20	SPTLC3	G	0.025	NA	-0.0137	0.0158	0.3850
LDL cholesterol	rs6029526	20	TOP1	A	0.044	NA	0.0084	0.0156	0.5902
LDL cholesterol	rs4253772	22	PPARA	T	0.031	NA	0.0090	0.0255	0.7240
LDL cholesterol	rs5763662	22	MTMR3	T	0.077	NA	0.0183	0.0508	0.7194
Triglycerides	rs12748152	1	PIGV-NR0B2	T	0.037	NA	0.0344	0.0295	0.2429
Triglycerides	rs2131925	1	ANGPTL3	T	0.066	NA	-0.0334	0.0164	0.04223
Triglycerides	rs4846914	1	GALNT2	G	0.040	NA	-0.0077	0.0166	0.6404
Triglycerides	rs1260326	2	GCKR	T	0.115	NA	-0.0008	0.0161	0.9608
Triglycerides	rs2972146	2	IRS1	T	0.028	NA	0.0131	0.0159	0.4116
Triglycerides	rs150213942	3	MSL2L1	G	0.029	NA	-0.0302	0.0184	0.1011
Triglycerides	rs442177	4	KLHL8	T	0.031	NA	0.0026	0.0159	0.8675
Triglycerides	rs6831256	4	LRPAP1	G	0.026	NA	0.0124	0.0157	0.4296
Triglycerides	rs6882076	5	TIMD4	C	0.029	NA	0.0084	0.0169	0.6190
Triglycerides	rs9686661	5	MAP3K1	T	0.038	NA	0.0263	0.0205	0.1993
Triglycerides	rs1936800	6	RSPO3	T	0.020	NA	0.0061	0.0157	0.6995
Triglycerides	rs998584	6	VEGFA	A	0.029	NA	-0.0292	0.0181	0.1061
Triglycerides	rs13238203	7	TYW1B	C	0.059	NA	0.0110	0.0663	0.8677
Triglycerides	rs17145738	7	MLXIPL	C	0.115	NA	-0.0255	0.0243	0.2943
Triglycerides	rs38855	7	MET	A	0.019	NA	-0.0048	0.0154	0.7554
Triglycerides	rs4722551	7	MIR148A	C	0.023	NA	-0.0221	0.0216	0.3072
Triglycerides	rs11776767	8	PINX1	C	0.022	NA	-0.0428	0.0159	0.0071
Triglycerides	rs12678919	8	LPL	A	0.170	NA	-0.0105	0.0251	0.6759
Triglycerides	rs1495741	8	NAT2	G	0.040	NA	-0.0041	0.0186	0.8269
Triglycerides	rs2954029	8	TRIB1	A	0.076	NA	0.0137	0.0160	0.3908
Triglycerides	rs10761731	10	JMJD1C	A	0.031	NA	0.0215	0.0156	0.1678
Triglycerides	rs1832007	10	AKR1C4	A	0.033	NA	0.0088	0.0216	0.6842
Triglycerides	rs2068888	10	CYP26A1	G	0.024	NA	0.0209	0.0162	0.1973
Triglycerides	rs174546	11	FADS1-2-3	T	0.045	NA	-0.0104	0.0168	0.5374
Triglycerides	rs964184	11	APOA1	G	0.234	NA	0.0207	0.0226	0.3617
Triglycerides	rs11613352	12	LRP1	C	0.028	NA	-0.0106	0.0185	0.5644
Triglycerides	rs4765127	12	ZNF664	G	0.029	NA	-0.0102	0.0161	0.5278
Triglycerides	rs1532085	15	LIPC	A	0.031	NA	-0.0390	0.0160	0.0148
Triglycerides	rs2412710	15	CAPN3	A	0.099	NA	-0.0276	0.0580	0.6348
Triglycerides	rs2929282	15	FRMD5	T	0.072	NA	-0.0339	0.0357	0.3422
Triglycerides	rs1121980	16	FTO	A	0.021	NA	0.0005	0.0157	0.9724
Triglycerides	rs11649653	16	CTF1	C	0.027	NA	-0.0407	0.0173	0.0184
Triglycerides	rs3198697	16	PDXDC1	C	0.020	NA	0.0152	0.0164	0.3527
Triglycerides	rs3764261	16	CETP	C	0.040	NA	-0.0032	0.0165	0.8462
Triglycerides	rs8077889	17	MPP3	C	0.025	NA	-0.0301	0.0196	0.1245
Triglycerides	rs10401969	19	CILP2	T	0.121	NA	0.0163	0.0346	0.6368
Triglycerides	rs7248104	19	INSR	G	0.022	NA	0.0111	0.0161	0.4912
Triglycerides	rs731839	19	PEPD	G	0.022	NA	0.0102	0.0168	0.5428
Triglycerides	rs6065906	20	PLTP	C	0.053	NA	-0.0153	0.0192	0.4275
Triglycerides	rs5756931	22	PLA2G6	T	0.020	NA	-0.0030	0.0161	0.8527

C-reactive protein	rs12037222	1	PABPC4	A	0.045	0.007	0.0175	0.0193	0.3640
C-reactive protein	rs12239046	1	NLRP3	C	0.047	0.006	-0.0006	0.0171	0.9726
C-reactive protein	rs2794520	1	CRP	C	0.160	0.006	0.0090	0.0168	0.5938
C-reactive protein	rs4129267	1	IL6R	C	0.079	0.005	-0.0006	0.0162	0.9683
C-reactive protein	rs4420065	1	LEPR	C	0.090	0.005	-0.0206	0.0164	0.2096
C-reactive protein	rs1260326	2	GCKR	T	0.072	0.005	-0.0008	0.0161	0.9608
C-reactive protein	rs6734238	2	IL1F10	G	0.050	0.006	0.0035	0.0156	0.8204
C-reactive protein	rs4705952	5	IRF1	G	0.042	0.007	0.0046	0.0197	0.8133
C-reactive protein	rs6901250	6	GPRC6A	A	0.035	0.006	0.0082	0.0171	0.6316
C-reactive protein	rs13233571	7	BCL7B	C	0.054	0.009	-0.0436	0.0273	0.1098
C-reactive protein	rs4240624	8	PPP1R3B	G	0.069	0.011	0.0130	0.0279	0.6399
C-reactive protein	rs10745954	12	ASCL1	A	0.039	0.006	0.0051	0.0155	0.7419
C-reactive protein	rs1183910	12	HNF1A	G	0.149	0.006	0.0173	0.0170	0.3083
C-reactive protein	rs340029	15	RORA	T	0.032	0.006	0.0268	0.0165	0.1043
C-reactive protein	rs10521222	16	SALL1	C	0.104	0.015	0.0364	0.0397	0.3589
C-reactive protein	rs2847281	18	PTPN2	A	0.031	0.006	0.0062	0.0158	0.6931
C-reactive protein	rs12721051	19	APOE/APOC1	C	0.236	0.009	-1.3043	0.0278	7.8×10^{-482}
C-reactive protein	rs1800961	20	HNF4A	C	0.088	0.015	-0.0596	0.0467	0.2016

BP = blood pressure; EA = effect allele; HDL = high-density lipoprotein; LDL = low-density lipoprotein; NA, not available; SE = standard error; SNP = single nucleotide polymorphism; WHRadjBMI = waist-to-hip ratio adjusted for body mass index. *Summary statistics from the International Genomics of Alzheimer's Project (17 008 Alzheimer's disease cases and 37 154 controls). †Effect size estimates (β coefficients) were obtained from published genome-wide association studies of the risk factors (see table S2) and represent one unit change in the risk factor per additional copy of the effect allele. The change is expressed in cigarettes per day for smoking, drinks per week for alcohol, cups per day for coffee, percentage for 25-hydroxyvitamin D, mmol/L for fasting glucose, ln-pmol/L for fasting insulin, mmHg for systolic and diastolic blood pressure, ln-mg/L for C-reactive protein, and standard deviation for the other continuous risk factors. For the binary risk factors, the change is expressed as log-odds of having the risk factor. In the Mendelian randomisation analyses, education was scaled to 1 year of education completed (1 SD = 3.6 years), smoking to 10 cigarettes, 25-hydroxyvitamin D to 20% change, fasting glucose to one SD (assuming one SD = 0.65 mmol/L), fasting insulin to one SD (assuming one SD = 0.60 ln-pmol/L), systolic and diastolic blood pressure to one SD (assuming one SD = 15 mmHg and 9.5 mmHg respectively), and C-reactive protein to one SD (assuming one SD = 1 ln-mg/L). The β coefficients for intelligence were estimated from the reported *z*-scores.

Table D. Association between genetically predicted one year increase in education completed and other modifiable factors*

Outcome	Estimate (95% CI)	P value
**Binary outcome**		
Ever smoked†	0.88 (0.84 to 0.92)	4.0×10^{-9}
**Continuous outcomes**		
Cigarettes per day†	-0.43 (-0.73 to -0.13) cig/day	0.005
Systolic blood pressure‡	-0.17 (-0.52 to 0.18) mmHg	0.35
Diastolic blood pressure‡	-0.02 (-0.20 to 0.25) mmHg	0.84
Low-density lipoprotein cholesterol§	-0.01 (-0.02 to 0.01) mmol/L	0.54
High-density lipoprotein cholesterol§	0.05 (0.035 to 0.07) mmol/L	1.4×10^{-9}
Total cholesterol§	0.00 (-0.02 to 0.02) mmol/L	0.87
Triglycerides§	-0.05 (-0.06 to -0.03) mmol/L	4.6×10^{-8}
Fasting glucose¶	0.00 (-0.15 to 0.01) mmol/L	0.78
Fasting insulin¶	-0.02 (-0.03 to -0.01) log pmol/L	0.004
Body mass index**	-0.29 (-0.33 to -0.19) kg/m^2	2.6×10^{-18}

*Analyses are based on 107 to 111 single nucleotide polymorphisms associated with education. †Data from the Tobacco and Genetics Consortium.[11] ‡Data from International Consortium for Blood Pressure (ICBP).[27] §Data from Global Lipids Genetics Consortium (GLGC).[22] ¶Data from Meta-Analyses of Glucose and Insulin-related traits Consortium (MAGIC).[26] **Data from Genetic Investigation of Anthropometric Traits consortium (GIANT).[17]

References

1 Xu W, Tan L, Wang HF, et al. Meta-analysis of modifiable risk factors for Alzheimer's disease. J Neurol Neurosurg Psychiatry 2015;86:1299-306. doi: 10.1136/jnnp-2015-310548

2 Liu QP, Wu YF, Cheng HY, et al. Habitual coffee consumption and risk of cognitive decline/dementia: A systematic review and meta-analysis of prospective cohort studies. Nutrition 2016;32:628-36. doi: 10.1016/j.nut.2015.11.015

3 Shen L, Ji HF. Vitamin D deficiency is associated with increased risk of Alzheimer's disease and dementia: evidence from meta-analysis. Nutr J 2015;14:76. doi: 10.1186/s12937-015-0063-7

4 Meng XF, Yu JT, Wang HF, et al. Midlife vascular risk factors and the risk of Alzheimer's disease: a systematic review and meta-analysis. J Alzheimers Dis 2014;42:1295-310. doi: 10.3233/JAD-140954

5 Koyama A, O'Brien J, Weuve J, et al. The role of peripheral inflammatory markers in dementia and Alzheimer's disease: a meta-analysis. J Gerontol A Biol Sci Med Sci 2013;68:433-40. doi: 10.1093/gerona/gls187

6 Feart C, Helmer C, Merle B, et al. Associations of lower vitamin D concentrations with cognitive decline and long-term risk of dementia and Alzheimer's disease in older adults. Alzheimers Dement 2017:13;1207-16. doi: 10.1016/j.jalz.2017.03.003

7 Olsson E, Byberg L, Karlstrom B, et al. Vitamin D is not associated with incident dementia or cognitive impairment: an 18-y follow-up study in community-living old men. Am J Clin Nutr 2017;105:936-43. doi: 10.3945/ajcn.116.141531

8 Okbay A, Beauchamp JP, Fontana MA, et al. Genome-wide association study identifies 74 loci associated with educational attainment. Nature 2016;533:539-42. doi: 10.1038/nature17671

9 Sniekers S, Stringer S, Watanabe K, et al. Genome-wide association meta-analysis of 78,308 individuals identifies new loci and genes influencing human intelligence. Nat Genet 2017;49:1107-12. doi: 10.1038/ng.3869

10 Thorgeirsson TE, Gudbjartsson DF, Surakka I, et al. Sequence variants at CHRNB3-CHRNA6 and CYP2A6 affect smoking behavior. Nat Genet 2010;42:448-53. doi: 10.1038/ng.573

11 Genome-wide meta-analyses identify multiple loci associated with smoking behavior. Nat Genet 2010;42:441-7. doi: 10.1038/ng.571

12 Jorgenson E, Thai KK, Hoffmann TJ, et al. Genetic contributors to variation in alcohol consumption vary by race/ethnicity in a large multi-ethnic genome-wide association study. Mol Psychiatry 2017;22:1359-67. doi: 10.1038/mp.2017.101

13 Cornelis MC, Byrne EM, Esko T, et al. Genome-wide meta-analysis identifies six novel loci associated with habitual coffee consumption. Mol Psychiatry 2015;20:647-56. doi: 10.1038/mp.2014.107

14 Vimaleswaran KS, Berry DJ, Lu C, et al. Causal relationship between obesity and vitamin D status: bi-directional Mendelian randomization analysis of multiple cohorts. PLoS Med 2013;10:e1001383. doi: 10.1371/journal.pmed.1001383

15 Grarup N, Sulem P, Sandholt CH, et al. Genetic architecture of vitamin B12 and folate levels uncovered applying deeply sequenced large datasets. PLoS Genet 2013;9:e1003530. doi: 10.1371/journal.pgen.1003530

16 van Meurs JB, Pare G, Schwartz SM, et al. Common genetic loci influencing plasma homocysteine concentrations and their effect on risk of coronary artery disease. Am J Clin Nutr 2013;98:668-76. doi: 10.3945/ajcn.112.044545

17 Locke AE, Kahali B, Berndt SI, et al. Genetic studies of body mass index yield new insights for obesity biology. Nature 2015;518:197-206. doi: 10.1038/nature14177

18 Shungin D, Winkler TW, Croteau-Chonka DC, et al. New genetic loci link adipose and insulin biology to body fat distribution. Nature 2015;518:187-96. doi: 10.1038/nature14132

19 Morris AP, Voight BF, Teslovich TM, et al. Large-scale association analysis provides insights into the genetic architecture and pathophysiology of type 2 diabetes. Nat Genet 2012;44:981-90. doi: 10.1038/ng.2383

20 Scott RA, Lagou V, Welch RP, et al. Large-scale association analyses identify new loci influencing glycemic traits and provide insight into the underlying biological pathways. Nat Genet 2012;44:991-1005. doi: 10.1038/ng.2385
21 Hoffmann TJ, Ehret GB, Nandakumar P, et al. Genome-wide association analyses using electronic health records identify new loci influencing blood pressure variation. Nat Genet 2017;49:54-64. doi: 10.1038/ng.3715
22 Willer CJ, Schmidt EM, Sengupta S, et al. Discovery and refinement of loci associated with lipid levels. Nat Genet 2013;45:1274-83. doi: 10.1038/ng.2797
23 Dehghan A, Dupuis J, Barbalic M, et al. Meta-analysis of genome-wide association studies in >80 000 subjects identifies multiple loci for C-reactive protein levels. Circulation 2011;123:731-8. doi: 10.1161/circulationaha.110.948570
24 Kong A, Steinthorsdottir V, Masson G, et al. Parental origin of sequence variants associated with complex diseases. Nature 2009;462:868-74. doi: 10.1038/nature08625
25 Voight BF, Scott LJ, Steinthorsdottir V, et al. Twelve type 2 diabetes susceptibility loci identified through large-scale association analysis. Nat Genet 2010;42:579-89. doi: 10.1038/ng.609
26 Dupuis J, Langenberg C, Prokopenko I, et al. New genetic loci implicated in fasting glucose homeostasis and their impact on type 2 diabetes risk. Nat Genet 2010;42:105-16. doi: 10.1038/ng.520
27 Ehret GB, Munroe PB, Rice KM, et al. Genetic variants in novel pathways influence blood pressure and cardiovascular disease risk. Nature 2011;478:103-9. doi: 10.1038/nature10405
