# Supplementary material for: Modifiable pathways in Alzheimer’s disease: Mendelian randomisation analysis
Source: BMJ. 2017 Dec 7;359:j5375. doi: 10.1136/bmj.j5375 (PMC5717765; doi:10.1136/bmj.j5375)
Supplement: Supplementary file 2 — Appendix 2: Supplementary figures A-J [file lars039122.ww2.pdf]

Appendix 2: Supplementary figures [posted as supplied by author]

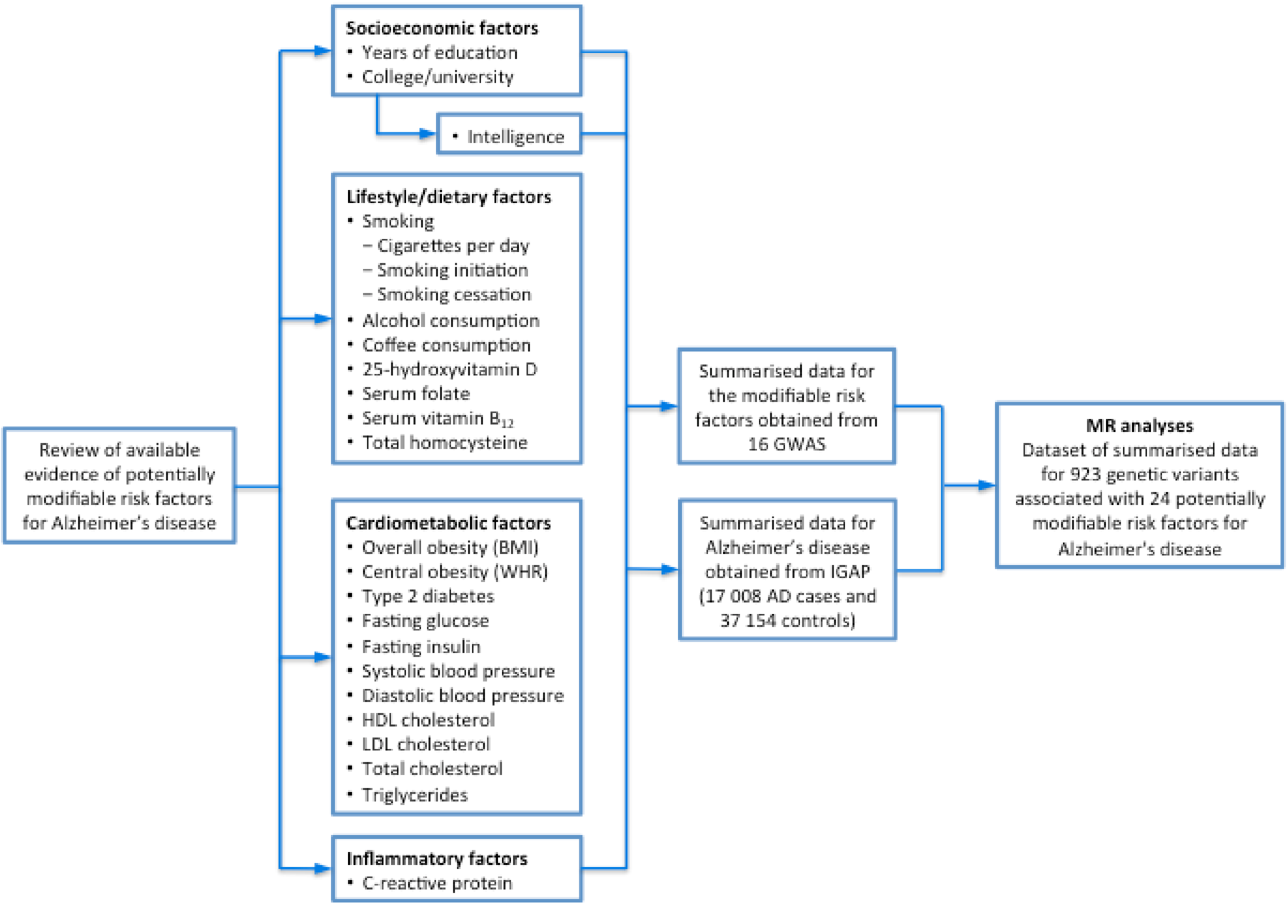

Figure A. Flowchart for deriving the dataset used for the Mendelian randomisation analyses. The 16 GWAS of the modifiable risk factors are described in table S2. BMI = body mass index; GWAS = genome-wide association studies; HDL = high-density lipoprotein; IGAP = International Genomics of Alzheimer's Project; LDL = low-density lipoprotein; MR = Mendelian randomisation; WHR = waist-to-hip ratio.

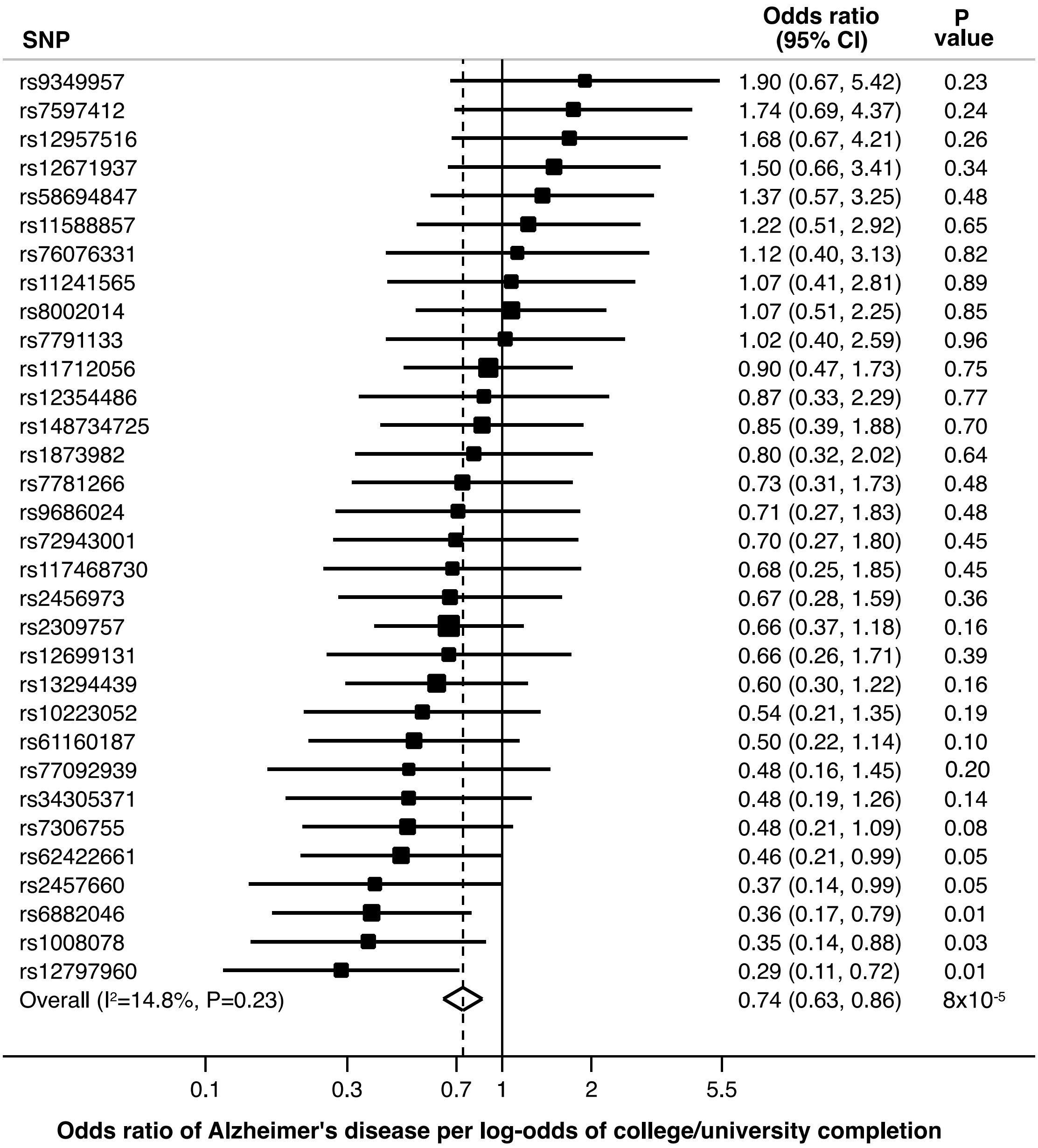

Figure B. Association between genetically predicted college/university completion and Alzheimer's disease. Squares represent the odds ratio of Alzheimer's disease (AD) per genetically predicted one unit increase in log-odds of having completed college/university; horizontal lines represent 95% confidence intervals (CIs); diamond represent the overall odds ratio with its 95% CI. SNP = single nucleotide polymorphism.

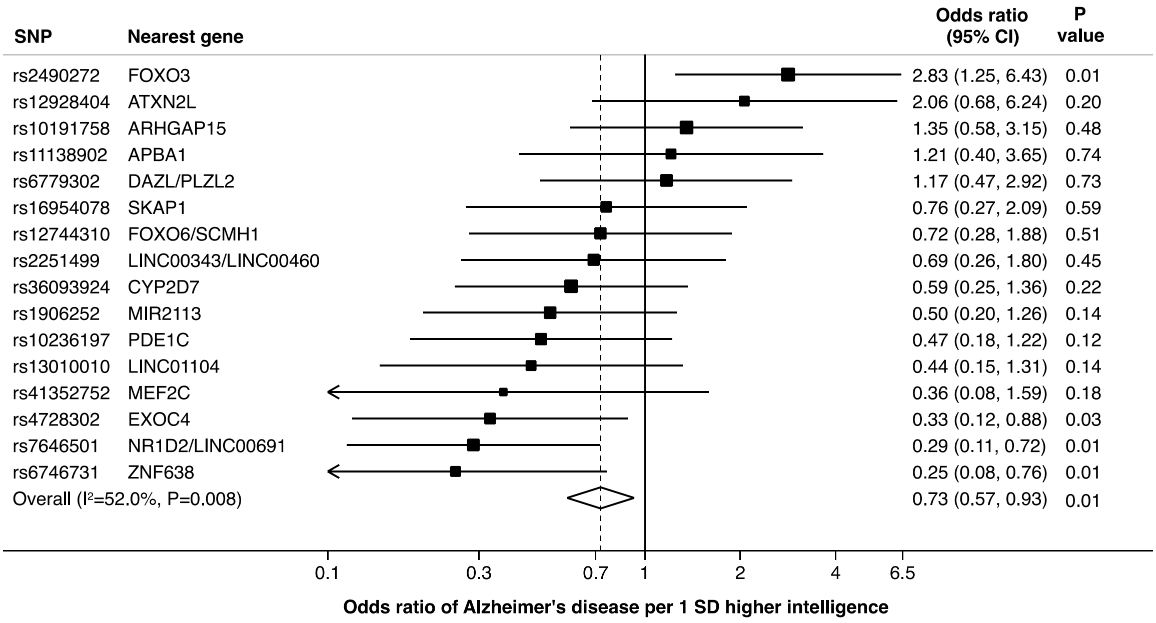

Figure C. Association between genetically predicted intelligence and Alzheimer's disease. Squares represent the odds ratio of Alzheimer's disease per genetically predicted one standard deviation increase in intelligence; horizontal lines represent 95% confidence intervals (CIs); diamond represent the overall odds ratio with its 95% CI. SNP = single nucleotide polymorphism.

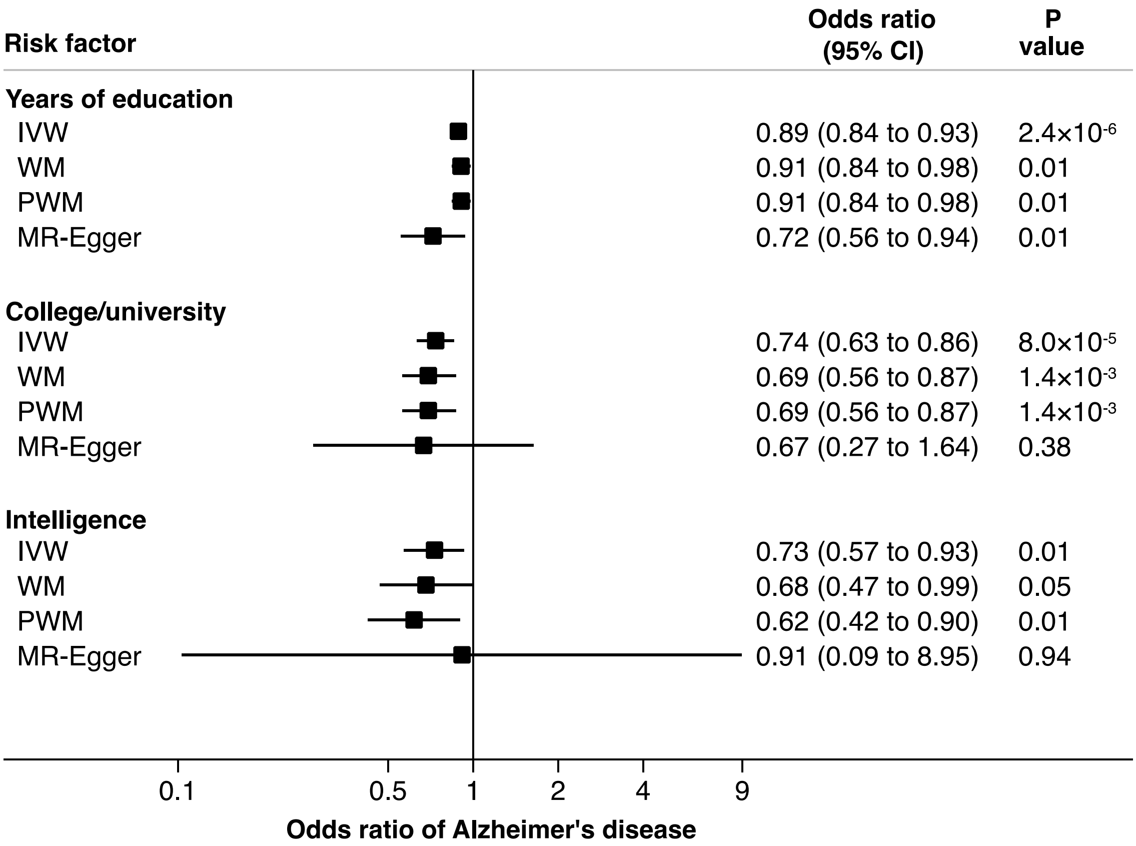

Figure D. Sensitivity analyses for the associations of genetically predicted years of education completed, college/university completion and intelligence with Alzheimer's disease, using four different Mendelian randomisation methods. IVW = inverse-variance weighted (conventional method); WM = weighted median; PWM = penalised weighted median (this method is a weighted median method that downweights the contribution to the analysis of genetic variants with outlying ratio estimates). The MR-Egger method did not detect evidence of directional pleiotropy (P=0.11 for years of education, P=0.83 for college/university, and P=0.84 for intelligence).

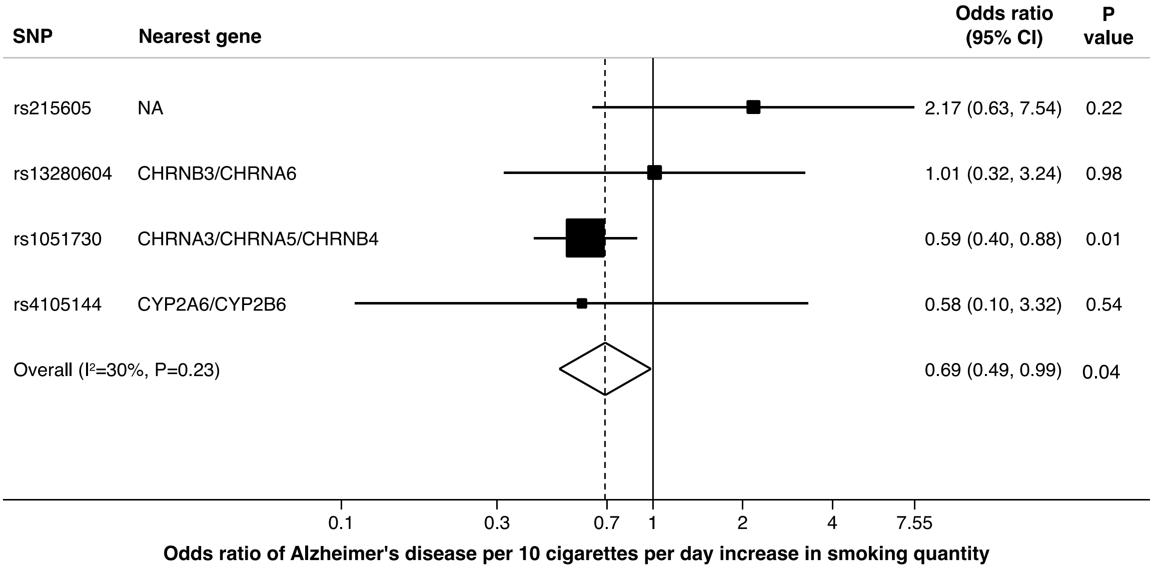

Figure E. Association between genetically predicted smoking and Alzheimer's disease. Squares represent the odds ratio of Alzheimer's disease per genetically predicted 10 cigarettes per day increase in smoking; horizontal lines represent 95% confidence intervals (CIs); diamond represent the overall odds ratio with its 95% CI. The genetic variant in or near the neuronal nicotinic acetylcholine receptor genes (CHRNA3/CHRNA5/CHRNB4) had a strong influence on the results and excluding this variant yielded an odds ratio of 1.21 (0.56 to 2.61; P=0.62). NA = not available; SNP = single nucleotide polymorphism.

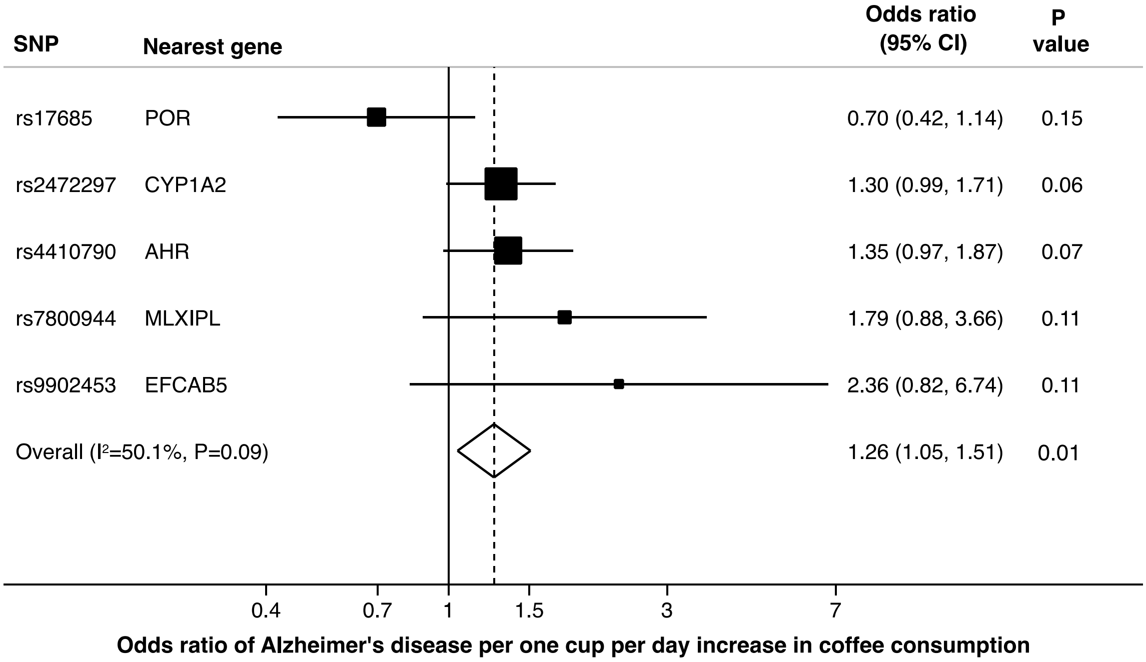

Figure F. Association between genetically predicted coffee consumption and Alzheimer’s disease. Squares represent the odds ratio of Alzheimer’s disease per genetically predicted one cup per day increase in coffee consumption; horizontal lines represent 95% confidence intervals (CIs); diamond represent the overall odds ratio with its 95% CI. SNP = single nucleotide polymorphism.

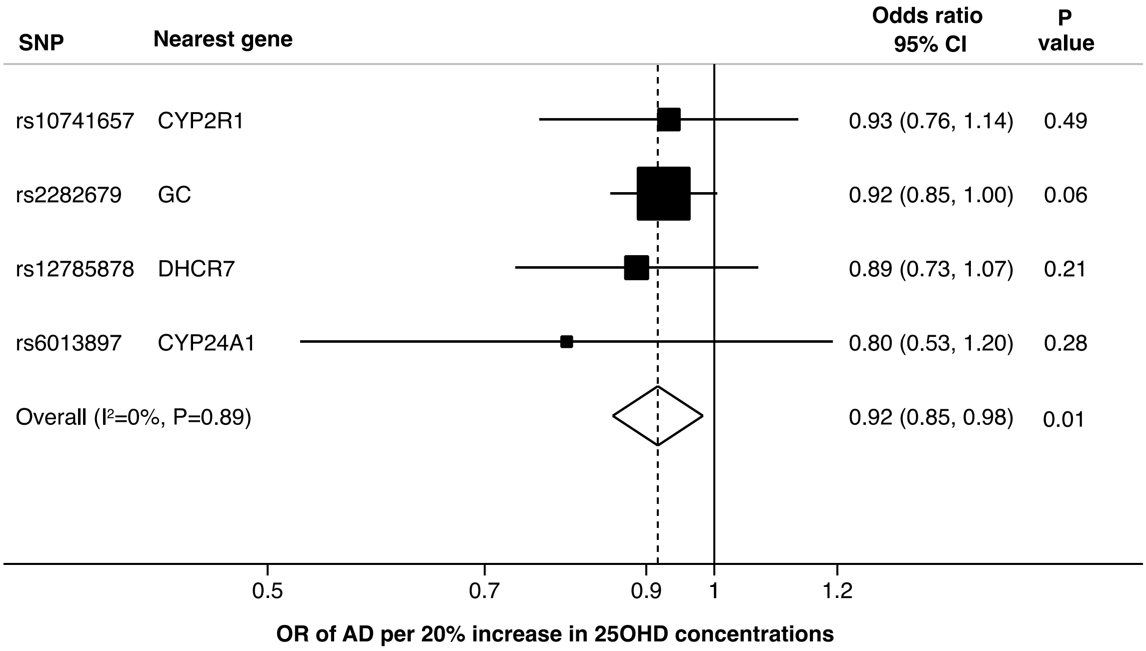

Figure G. Association between genetically predicted 25-hydroxyvitamin D and Alzheimer's disease. Squares represent the odds ratio of Alzheimer's disease per genetically predicted 20% increase in 25-hydroxyvitamin D (25OHD); horizontal lines represent 95% confidence intervals (CIs); diamond represent the overall odds ratio with its 95% CI. SNP = single nucleotide polymorphism.

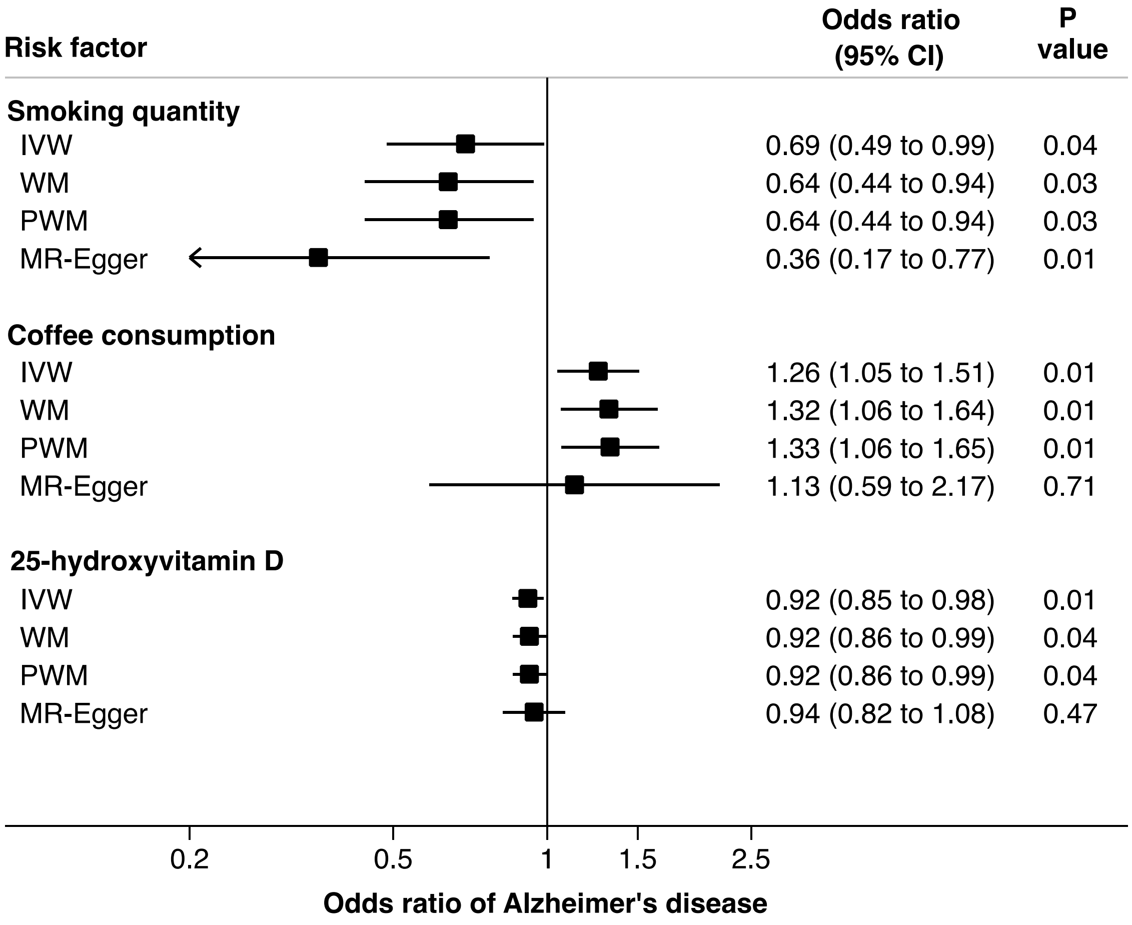

Figure H. Sensitivity analyses of the associations of genetically predicted smoking quantity, coffee consumption, and 25-hydroxyvitamin D with Alzheimer's disease, using four different Mendelian randomisation methods. IVW = inverse-variance weighted (conventional method); WM = weighted median; PWM = penalised weighted median (this method is a weighted median method that downweights the contribution to the analysis of genetic variants with outlying ratio estimates). The MR-Egger method did not detect evidence of directional pleiotropy (P=0.002 for smoking, P=0.72 for coffee, and P=0.17 for 25-hydroxyvitamin D).

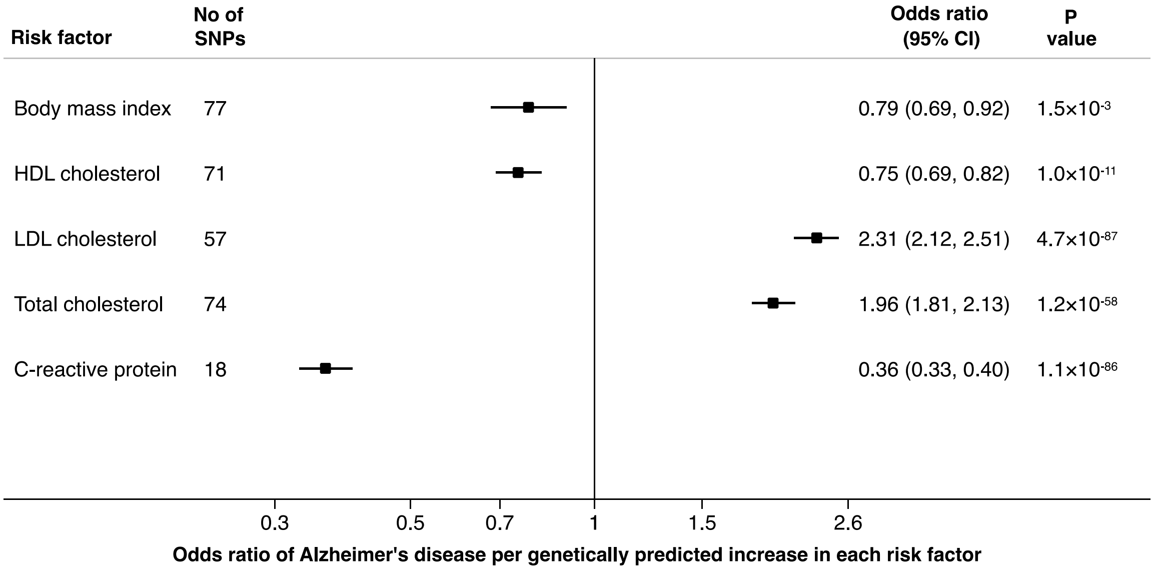

Figure I. Associations of genetically predicted body mass index, high-density lipoprotein cholesterol, low-density lipoprotein cholesterol, total cholesterol, and C-reactive protein with Alzheimer's disease in analyses including the pleiotropic genetic variant near the *APOE* gene, which is strongly associated with Alzheimer's disease. HDL = high-density lipoprotein; LDL = low-density lipoprotein; SNP = single nucleotide polymorphism.

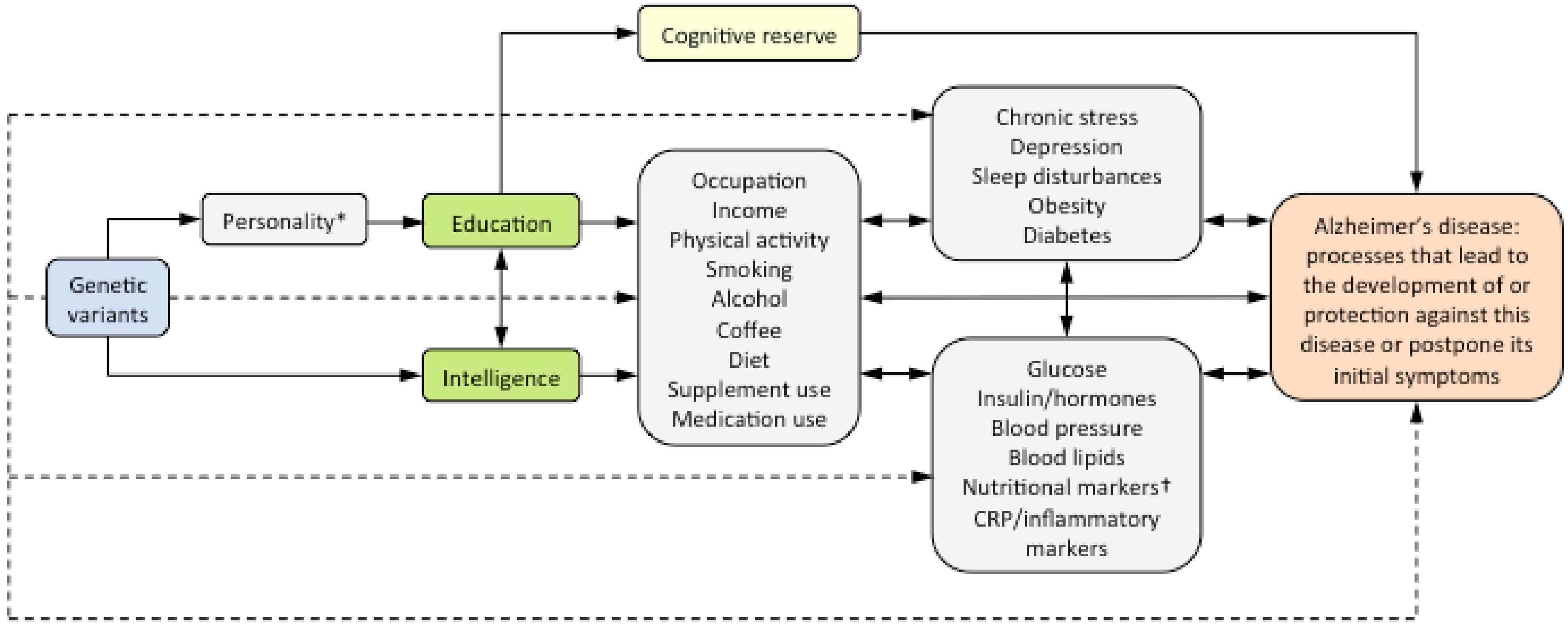

Figure J. Simplified diagram of potential pathways linking genetic variants associated with educational attainment and intelligence to these traits and to potential mediators and Alzheimer's disease. The dashed line represents pathways that would invalidate a Mendelian randomisation analysis of education and intelligence in relation to risk ofAlzheimer's disease. *For example self-discipline, organisational skills, better study skills, and brain function. †Nutrients and other compounds obtained from the diet or dietary supplements (eg, 25-hydroxyvitamin D [also determined to a large extent by sun exposure], folate, vitamin B_{12}, and homocysteine [determined to a large extent by dietary and lifestyle factors, such as folate and B_{12} levels, smoking, and coffee consumption]), which were examined for an association with Alzheimer's disease in the present Mendelian randomisation analysis. CRP = C-reactive protein.
