# Supplementary material for: Modifiable pathways in Alzheimer’s disease: Mendelian randomisation analysis
Source: BMJ. 2017 Dec 7;359:j5375. doi: 10.1136/bmj.j5375 (PMC5717765; doi:10.1136/bmj.j5375)
Supplement: Supplementary file 3 — Appendix 3: Description of the original genetic analyses and diagnostic assessment [file lars039122.ww3.pdf]

Appendix 3: IGAP – Description of the original genetic analyses and diagnostic assessment [posted as supplied by author]

International Genomics of Alzheimer's Project (IGAP) is a large two-stage study based upon genome-wide association studies (GWAS) on individuals of European ancestry.[1] In stage 1, IGAP used genotyped and imputed data on 7,055,881 single nucleotide polymorphisms to meta-analyse four GWAS datasets with a total of 17 008 Alzheimer's disease cases and 37 154 controls (The Alzheimer Disease Genetics Consortium [ADGC], The Cohorts for Heart and Aging Research in Genomic Epidemiology consortium [CHARGE], The European Alzheimer's disease Initiative [EADI], and The Genetic and Environmental Risk in AD consortium [GERAD]).

Analyses. In each case-control dataset, the association between genotype dose and AD was analysed by logistic regression and adjusted for age, sex, and principal components.[1] For the three CHARGE cohorts with incident Alzheimer's disease cases, the analyses were conducted using Cox proportional hazards regression models. Summary statistics across the datasets were combined using fixed-effects inverse variance-weighted meta-analysis with the standard errors of the beta-coefficients scaled by the square roots of the study-specific genomic inflation factors.[1]

Diagnostic criteria for Alzheimer's disease in each study/consortium

	**Alzheimer's disease cases**	
**Consortium**	**Cases**	**Diagnostic criteria**
**ADGC**		
ACT	566	NINCDS-ADRDA criteria for possible or probable AD
ADC	2512	DSM-IV criteria or CDR ≥1 (70% autopsy-confirmed)
ADNI	268	NINCDS-ADRDA criteria for probable AD
GenADA	669	NINCDS-ADRDA and DSM-IV criteria for probable AD
Mayo-Clinic	728	NINCDS-ADRDA for possible or probable AD (34% autopsy-confirmed)
MIRAGE	509	NINCDS-ADRDA criteria for probable or definite AD
NCRAD/NIA-LOAD	1811	NINCDS-ADRDA criteria for possible, probable or definite AD
OHSU	131	Autopsy-confirmed AD
ROS/MAP	291	Clinical criteria for AD
TGEN2	129	Possible or probable AD (autopsy-confirmed)
UM/VU/MSSM	1070	NINCDS-ADRDA for probable or definite AD (34% autopsy-confirmed)
UP	1271	NINCDS-ADRDA criteria for probable or definite AD
WU	318	Standard criteria
**CHARGE**		
AGES-RS	78	NINCDS-ADRDA criteria for possible or probable AD
CHS	421	NINCDS-ADRDA criteria for possible or probable AD
FHS	183	NINCDS-ADRDA criteria for possible, probable or definite AD
Rotterdam study	633	NINCDS-ADRDA criteria for possible, probable or definite AD
**EADI**	2243	NINCDS-ADRDA criteria for probable AD
**GERAD**	3177	NINCDS-ADRDA and DSM-IV criteria for probable AD and CERAD criteria for definite AD

AD = Alzheimer's disease; CDR = Clinical Dementia Rating; CERAD = Consortium to Establish a Registry for Alzheimer's Disease; DSM-IV = Diagnostic and Statistical Manual of Mental Disorders, 4th Edition; NINCDS-ADRDA = National Institute of Neurological and Communicative Disorders and Stroke and the Alzheimer's Disease and Related Disorders Association.

Reference

1 Lambert JC, Ibrahim-Verbaas CA, Harold D, et al. Meta-analysis of 74,046 individuals identifies 11 new susceptibility loci for Alzheimer's disease. Nat Genet 2013;45:1452-8. doi: 10.1038/ng.2802
