# Supplementary material for: Modifiable pathways in Alzheimer’s disease: Mendelian randomisation analysis
Source: BMJ. 2017 Dec 7;359:j5375. doi: 10.1136/bmj.j5375 (PMC5717765; doi:10.1136/bmj.j5375)
Supplement: Supplementary file 4 — Appendix 4: Further details of contribution from the International Genomics of Alzheimer’s Project [file lars039122.ww4.pdf]

Appendix 4: Further details of contribution from the International Genomics of Alzheimer's Project [posted as supplied by author]

The investigators within International Genomics of Alzheimer's Project (IGAP) contributed to the design and implementation of IGAP and/or provided data but did not participate in analysis or writing of this report. IGAP was made possible by the generous participation of the control subjects, the patients, and their families. The i–Select chips was funded by the French National Foundation on Alzheimer's disease and related disorders. EADI was supported by the LABEX (laboratory of excellence program investment for the future) DISTALZ grant, Inserm, Institut Pasteur de Lille, Université de Lille 2 and the Lille University Hospital. GERAD was supported by the Medical Research Council (Grant n° 503480), Alzheimer's Research UK (Grant n° 503176), the Wellcome Trust (Grant n° 082604/2/07/Z) and German Federal Ministry of Education and Research (BMBF): Competence Network Dementia (CND) grant n° 01GI0102, 01GI0711, 01GI0420. CHARGE was partly supported by the NIH/NIA grant R01 AG033193 and the NIA AG081220 and AGES contract N01–AG–12100, the NHLBI grant R01 HL105756, the Icelandic Heart Association, and the Erasmus Medical Center and Erasmus University. ADGC was supported by the NIH/NIA grants: U01 AG032984, U24 AG021886, U01 AG016976, and the Alzheimer's Association grant ADGC–10–196728.
